# OneSC: a computational platform for recapitulating cell state transitions

**DOI:** 10.1093/bioinformatics/btae703

**Published:** 2024-11-21

**Authors:** Da Peng, Patrick Cahan

**Affiliations:** Department of Biomedical Engineering, Johns Hopkins University, Baltimore, MD 21205, United States; Department of Biomedical Engineering, Johns Hopkins University, Baltimore, MD 21205, United States; Institute for Cell Engineering, Johns Hopkins University, Baltimore, MD 21205, United States; Department of Molecular Biology and Genetics, Johns Hopkins University, Baltimore, MD 21205, United States

## Abstract

**Motivation:**

Computational modeling of cell state transitions has been a great interest of many in the field of developmental biology, cancer biology, and cell fate engineering because it enables performing perturbation experiments *in silico* more rapidly and cheaply than could be achieved in a lab. Recent advancements in single-cell RNA-sequencing (scRNA-seq) allow the capture of high-resolution snapshots of cell states as they transition along temporal trajectories. Using these high-throughput datasets, we can train computational models to generate *in silico* “synthetic” cells that faithfully mimic the temporal trajectories.

**Results:**

Here we present OneSC, a platform that can simulate cell state transitions using systems of stochastic differential equations govern by a regulatory network of core transcription factors (TFs). Different from many current network inference methods, OneSC prioritizes on generating Boolean network that produces faithful cell state transitions and terminal cell states that mimic real biological systems. Applying OneSC to real data, we inferred a core TF network using a mouse myeloid progenitor scRNA-seq dataset and showed that the dynamical simulations of that network generate synthetic single-cell expression profiles that faithfully recapitulate the four myeloid differentiation trajectories going into differentiated cell states (erythrocytes, megakaryocytes, granulocytes, and monocytes). Finally, through the *in silico* perturbations of the mouse myeloid progenitor core network, we showed that OneSC can accurately predict cell fate decision biases of TF perturbations that closely match with previous experimental observations.

**Availability and implementation:**

OneSC is implemented as a Python package on GitHub (https://github.com/CahanLab/oneSC) and on Zenodo (https://zenodo.org/records/14052421).

## 1 Introduction

Cell fate decisions are governed, in part, by the set of interactions between transcription factors (TFs) and their target genes, which collectively form gene regulatory networks (GRNs) (Karlebach and Shamir 2008, Moignard *et al.* 2015, Churko *et al.* 2018, Almeida *et al.* 2021, Cahan *et al.* 2021). Inferring GRNs and using them to model cell behavior is widespread in development biology (Su *et al.* 2022a, Alanis-Lobato *et al.* 2024), cancer biology (Seçilmiş *et al.* 2020, Keyl *et al.* 2023), drug development (Alvarez *et al.* 2018), and cell fate engineering (Cahan *et al.* 2014, Rackham *et al.* 2016, Hartmann *et al.* 2018). There are three commonly used yet distinct approaches to reconstructing GRNs. In one approach, the set of genes bound and potentially regulated by a TF, its regulons, is determined with methods such as ChIP-seq (Park 2009) and CUT&Tag (Kaya-Okur *et al.* 2019). This approach is feasible when studying biological systems comprised of relatively few TFs for which antibodies are available. A second approach infers GRNs by mapping TF binding site motifs to accessible chromatin (Song and Crawford 2010, Buenrostro *et al.* 2013) in cis regulatory elements of putative target genes (Pranzatelli *et al.* 2018, Yan *et al.* 2020). A third approach is to infer GRNs genomewide based on the assumption that statistical association between TF and putative target gene expression implies a regulatory interaction, which can be achieved using bulk (Marbach *et al.* 2012) and single-cell RNA-sequencing (scRNA-seq) data (Pratapa *et al.* 2020, Nguyen *et al.* 2021). The relative merits of these methodologies and efforts to integrate them have been reviewed elsewhere (Badia-I-Mompel *et al.* 2023, Kim *et al.* 2023).

In this study, we focus specifically on inferring GRNs from scRNA-seq in such a way to maximize the fidelity of GRN-generated expression states in comparison to real transcriptional states. Currently, there are many methods to infer GRNs from single-cell expression profiles. These methods can largely be grouped into several broad categories based on the main algorithms: tree-based methods (GENIE3, Huynh-Thu *et al.* 2010; GRNBoost2, Moerman *et al.* 2019), correlation or information theory-based methods (PPCOR, Kim 2015; Epoch, Su *et al.* 2022a; LEAP, Specht and Li 2017; PIDC, Chan *et al.* 2017; SCRIBE, Qiu *et al.* 2020), regression-based methods (SINCERITIES, Papili Gao *et al.* 2018; SINGE, Deshpande *et al.* 2022), differential equation-based methods (SCODE, Matsumoto *et al.* 2017; GRISLI, Aubin-Frankowski and Vert 2020), and Bayesian network-based method (GRNVBEM, Sanchez-Castillo *et al.* 2018). GRNs are typically evaluated based on regulatory edge recovery (precision and recall) rather than the extent to which inferred GRNs can operate as a functional dynamical system (i.e. can the GRN model simulate certain biological phenomena such as cell differentiation?). Here, we define functional GRNs as those with the following two properties: (1) capable of generating rich dynamical behaviors that reflect biologically relevant steady states (Guantes and Poyatos 2008, Ye *et al.* 2019, Heydari *et al.* 2022, Huang *et al.* 2022, Su *et al.* 2022b) and (2) capable of generating perturbation predictions (Heydari *et al.* 2022, Su *et al.* 2022b).

Boolean networks can satisfy these conditions, and have successfully been used to model a wide range of biological phenomena such as embryonic stem cell self-renewal (Dunn *et al.* 2019), transitions between pluripotent states (Yachie-Kinoshita *et al.* 2018), T-cell development (Heydari *et al.* 2022, Ildefonso and Finley 2023), and *Drosophila melanogaster* segment polarity (Parmer *et al.* 2022). Previously RE: IN was developed to synthesize Abstract Boolean Networks (a set of concrete Boolean networks) that are consistent with experimental constraints and was used to identify the TF circuit for modeling naïve pluripotency (Dunn *et al.* 2014). More recently, IQCELL used satisfiability modulo theories engine (Z3) (de Moura and Bjørner 2008), a computational method that was also used in RE: IN (Dunn *et al.* 2014, Moignard *et al.* 2015, Hamey *et al.* 2017), to identify Boolean logic functions that are constrained by the pseudotime dynamics of binarized gene expressions (Heydari *et al.* 2022). The authors have demonstrated IQCELL’s capabilities of constructing Boolean networks that model early mouse T-cell and red blood cells development. Although simple, Boolean networks are an attractive method to study functional gene regulation because they capture the essential sigmoidal step function for characterizing concentration levels in many regulatory processes (Schwab *et al.* 2020) even in the absence of difficult-to-determine kinetic parameters (Barbuti *et al.* 2020). Simulating changes in Boolean network states is also computationally straightforward (Wang *et al.* 2012, Schwab *et al.* 2020) and thus facilitates performing dynamical simulations that produce testable predictions (Wang *et al.* 2012, Schwab *et al.* 2020). However, generating a functional Boolean network that produces attractor states mimicking the real data is not a trivial feat. A previous benchmark of a dozen methods that infer GRNs from single-cell expression profiles (Pratapa *et al.* 2020) showed that many of the methods, while not designed specifically for inferring functional Boolean networks, cannot produce Boolean networks with high simulation fidelity that exhibit the same number of steady states identical to those of the gold standard networks.

Here, we present *One* tool to *S*imulate *C*ells (OneSC), a computational platform to simulate cell state transitions observed in single-cell expression data using a system of stochastic differential equations guided by an inferred functional GRN. BoolODE (Pratapa *et al.* 2020) has previously demonstrated the feasibility of simulating realistic synthetic single-cell expression profiles across developmental trajectories using a GRN and a system of stochastic differential equations that fundamentally represent a set of predefined Boolean logic for transcriptional regulations. Extending the idea of simulating synthetic single-cell expression profiles using a functional GRN, OneSC has two main components: (1) a more scalable computational method to simulate single-cell expression profiles like those of BoolODE and (2) a computational method to infer a GRN that maximizes the fidelity of GRN-generated expression states in comparison to real transcriptional states. Like BoolODE, OneSC also uses stochastic differential equations to simulate expressions of genes governed by transcriptional regulations from a network. Improving upon BoolODE, OneSC’s simplified stochastic differential equations to model regulation allows OneSC to be more scalable and faster when simulating dense networks, and OneSC allows for native perturbation functionality.

In the other part of OneSC, we focus specifically on inferring GRNs from scRNA-seq data in such a way to maximize the fidelity of GRN-generated expression states in comparison to real transcriptional states using the OneSC simulator. Previously, a landmark benchmark study (using BoolODE and the BEELINE platform) reported that networks inferred from state-of-the art inference methods suffered from low simulation fidelity and were unable to generate the same number of steady states as found in the ground truth networks (Pratapa *et al.* 2020). To address this, our primary goal with OneSC was to improve upon the low simulation fidelity issue of current GRN inference methods. Our approach was to use additional information from standard scRNA-seq processing results (Luecken and Theis 2019) such as cell clusters and pseudotime annotations, in addition to gene-level expression estimates, to construct Boolean networks that capture the cell state transitions and generate the attractor states that match the terminal cell types observed in the single-cell dataset.

OneSC has two notable features that distinguish it from the recently published method IQCELL (Heydari *et al.* 2022). First, GRN inference is applied to all cell states in the dataset across all trajectories. This allows OneSC to generate one GRN that encapsulates branching processes (i.e. bifurcation or trifurcation) instead of needing to infer trajectory specific GRNs. Second, OneSC simulation platform allows for more continuous simulation of gene expressions than discrete asynchronous Boolean update. The main advantage to the continuous simulation of gene expressions is the expansion of gene state spaces beyond binary (on/off) through modeling partial on or partial off of genes, which was previously shown to generate cell states that closely match real transcriptional states in both space and time (Kaul *et al.* 2023).

In this work, we benchmarked OneSC’s GRN inference performance with over a dozen of current GRN inference methods using synthetic data on BEELINE platform (Pratapa *et al.* 2020), finding that OneSC networks have higher F1 scores. More importantly, we find that simulations performed with OneSC-generated networks achieve a higher similarity to real data. We also find that OneSC’s stochastic simulation platform, though very similar to BoolODE, is more computationally scalable. To illustrate how OneSC could be used to derive biological insights, we applied it to real single-cell expression data of mouse myeloid progenitor cells (Paul *et al.* 2015). This analysis produced a functional Boolean network that recaptured normal myelopoietic differentiation trajectories, and *in silico* perturbations resulted in cell fate decision biases that largely match the results of *in vivo* and *in vitro* genetic perturbations. In conclusion, we show that OneSC, coupling GRN inference and network simulation functionalities, is a useful tool to model differentiation and predict consequences of TF perturbations.

## 2 Materials and methods

**Figure 1.**
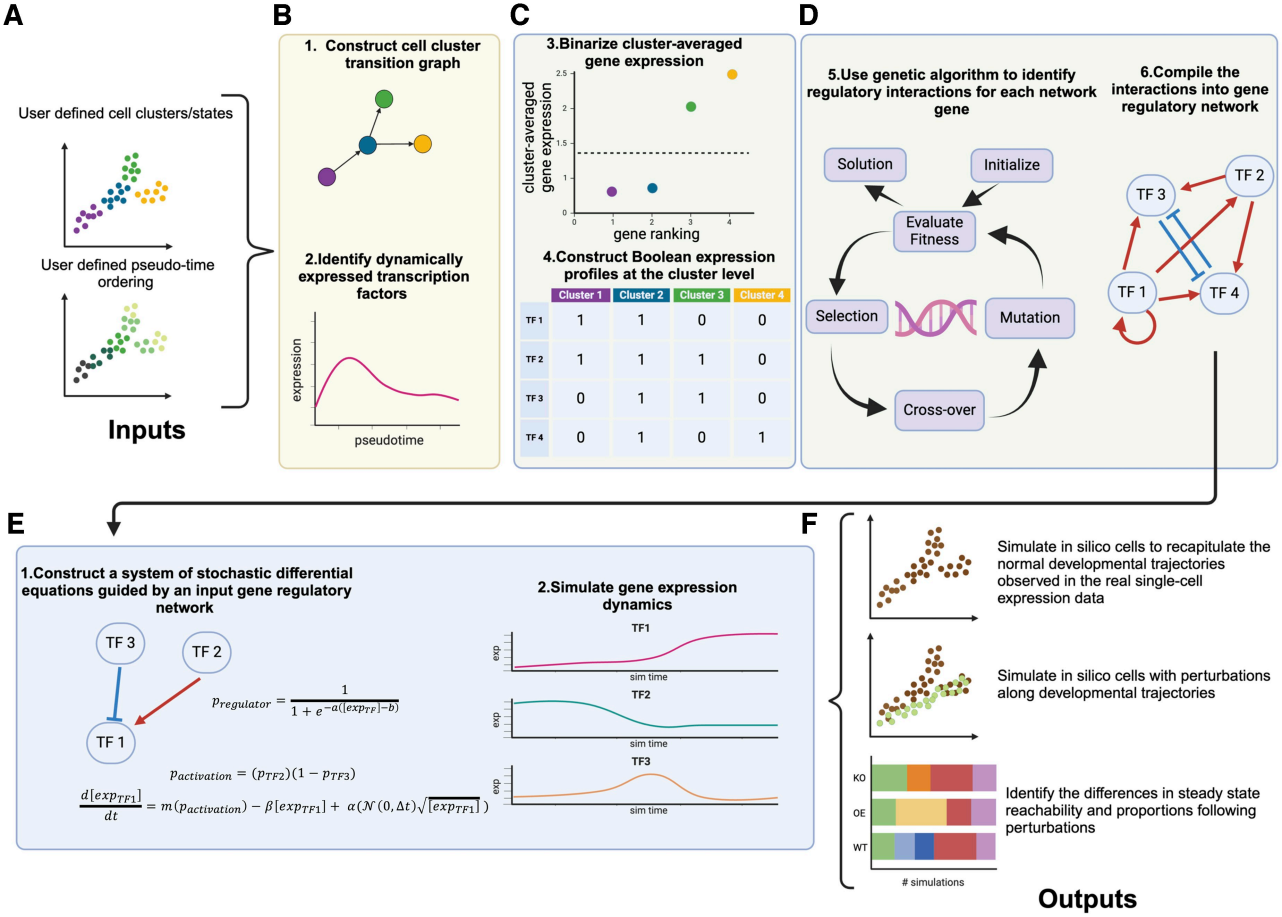
Overview of OneSC’s workflow. (A) Using the single-cell expression profiles and the inputted cell type cluster annotations with pseudotime information. (B) OneSC first constructs the cell cluster transition graph summarizing the cell state transitions observed in the single-cell data. From the individual trajectories in the cell state transition graph, OneSC next identifies dynamically expressed transcription factors for the construction of the gene regulatory network. Users can also manually create the cluster transition graph and select a set of transcription factors without using OneSC’s built-in functions. (C) Then, OneSC averages the gene expression profiles for each cell type cluster and binarizes them into activity status (1 or 0 representing on or off). (D) For each gene in the network, OneSC uses genetic algorithm to identify a set of regulatory interactions between target gene and its regulators such that the agreement between the observed activity status of the target gene and the simulated activity status across all cell states is maximized. Lastly, OneSC compiles all the subnetworks for individual target genes into a large network. (E) Using the inferred network, OneSC constructs a system of stochastic differential equations to simulate the gene dynamics. (F) Coupling the network inference and simulation, OneSC can generate simulated cells during normal cell state transitions or under perturbations to predict the shift in steady-state reachability and proportion. This figure was created using BioRender.com.

The first major function of OneSC is to infer GRNs from scRNA-seq data. OneSC requires three minimal inputs to perform this task: normalized single-cell expression profiles, cluster/cell type annotations, and pseudotime assignments (Fig. 1A). Given these, OneSC first constructs a cell cluster/state transition graph that reflects the relationship between clusters based on pseudotemporal ordering and expression profile similarity (Fig. 1B and Supplementary Info S1A). Second, OneSC identifies a core set of dynamically expressed TFs in each trajectory based on several criteria. First, OneSC fits a generalized additive model to predict gene expression based on pseudotime. It then selects the TFs that have significant (adjusted *P*-value<0.05 by default) smooth term for pseudotime (Su *et al.* 2022a) (Fig. 1B and Supplementary Info S1B). This suggests the relationship between gene expression and pseudotime is statistically significant. Then, OneSC further selects the dynamically expressed TFs that are sufficiently expressed in at least one cluster (percent expression of 0.1 by default) and have sufficient amount of difference between the highest expressing cluster and the lowest expressing cluster (log-fold change of 2 by default). It is important to note that users can also construct cell state transition graphs and select important TFs manually, independent of OneSC’s helper functions or they can modify upon the results obtained from OneSC’s helper functions. Third, OneSC averages the expression of dynamically expressed TFs in each cell type cluster and binarizes them into activity status: 1 or 0 (on or off) resulting in Boolean expression profiles (Fig. 1C and Supplementary Info S1C). Fourth, OneSC uses genetic algorithm (GA) (Mirjalili 2019), an evolutionary inspired metaheuristic optimization process that has previously been used in GRN inference (Chen *et al.* 2015, Barman and Kwon 2018, Trinh and Kwon 2021, Zhang *et al.* 2021) and refinement (Park *et al.* 2023), to identify regulatory interactions (i.e. activation, repression, or no regulation) between the potential regulators and the target gene (Fig. 1D and Supplementary Info S1D). GA’s ability to perform derivative-free optimization and to avoid local minima (Kramer *et al.* 2011) makes it an attractive optimization algorithm for network inference. The goal of GA here is to identify a subnetwork of direct regulators that maximizes the agreement between simulated activity status of the target gene (driven by the subnetwork configuration and activity status of the direct regulators) and the activity status observed in all real cell states (across all trajectories if there are branching processes). Lastly, OneSC compiles all the inferred subnetworks for each target gene into one large functional GRN (Fig. 1D).

The second major function of OneSC is to simulate transcriptional states from the previously inferred GRN and an initial state activity status profile. Alternatively, OneSC offers the flexibility for users to use their own curated GRNs and simulate them if the GRN conforms to the constraint that all nodes in the network have at least one regulator (including self-activator). OneSC generates single-cell expression profiles with systems of stochastic differential equations that model the expression dynamics of each gene (Fig. 1E and Supplementary Info S2). The expression value of target gene is regulated by the activity status of upstream TFs through a set of Boolean algebra (Shannon 1938). With the use of Boolean algebra and differential equations, OneSC dynamically simulates continuous expression values between simple binary values (on and off) to overcome the limitation of binary output of asynchronous or synchronous Boolean updates (Barbuti *et al.* 2020). OneSC then generates synthetic expression profiles across simulation that mimic pseudotemporal trajectories (Fig. 1F). Furthermore, OneSC can natively perform *in silico* overexpression and knockout to explore how these perturbations impact trajectories, the reachability of terminal states, and cell type/cluster compositions (Fig. 1F).

## 3 Results

### 3.1 OneSC accurately infers faithful GRNs

**Figure 2.**
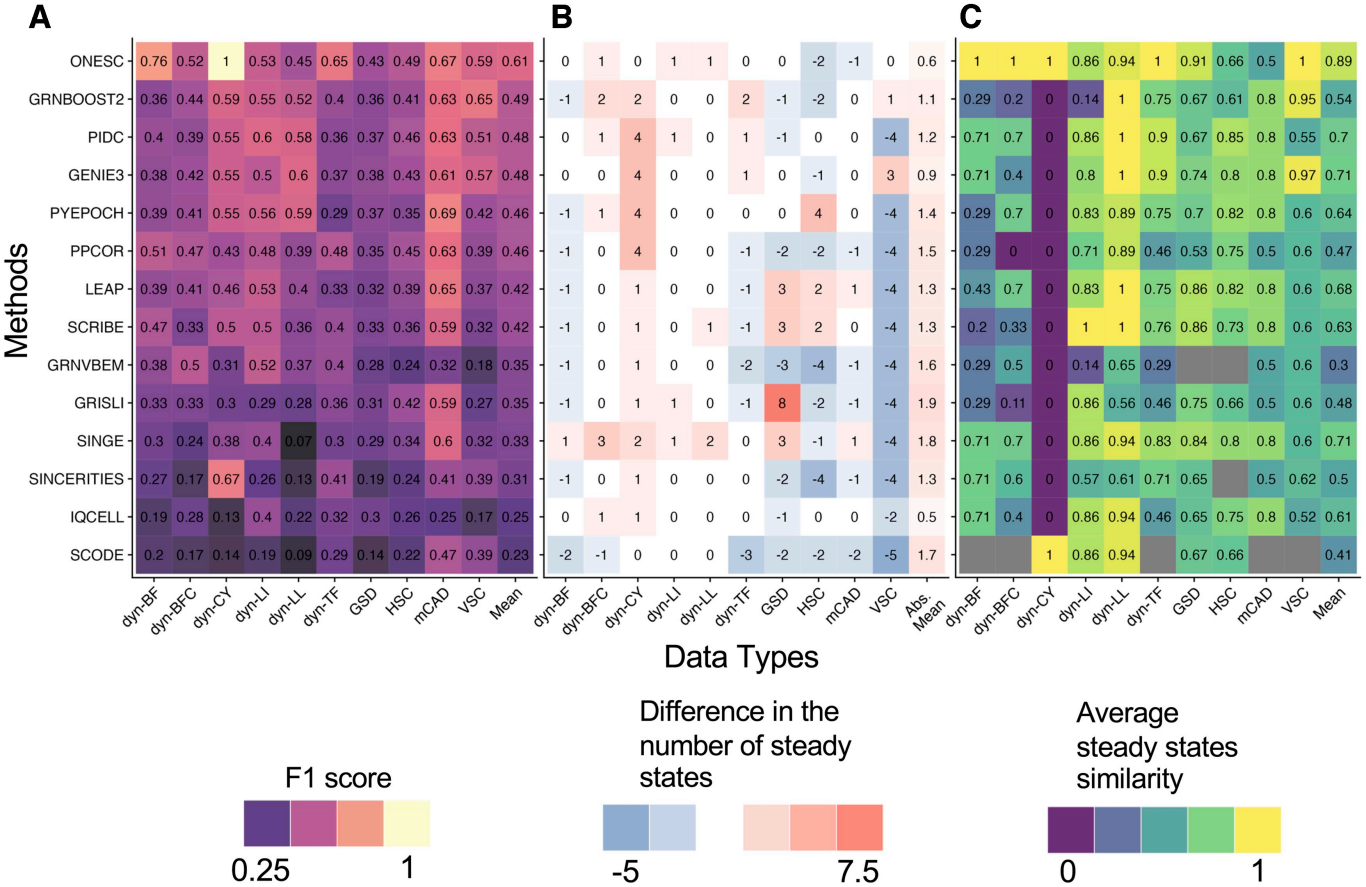
Benchmarking OneSC’s gene regulatory network inference method with other methods using BEELINE platform. (A) Heatmap showing the maximum F1 scores of various GRN inference methods tested on 10 synthetic datasets with associated gold standard networks. (B) Heatmap showing the differences in the number of steady states generated by the inferred GRNs and gold standard networks using asynchronous Boolean updates. (C) Heatmap of the average similarity scores between activity profiles of the steady states generated from inferred networks and those from gold standard networks using asynchronous Boolean updates. Similarity score is calculated as the proportion of genes that match Boolean activity status between two steady states. For each steady state in the gold standard network, the highest similarity score between it and all the steady states from inferred GRN is found. The maximum similarity scores for all gold standard steady states are averaged to represent the overall simulation fidelity between inferred GRNs and gold standard networks. In the dyn-CY dataset the average steady state similarity score is assigned a zero if the inferred network has at least one steady state and a one otherwise because dyn-CY gold standard network does not have a steady state. The gray color box indicates that no steady state was found in the inferred network.

Simulation of accurate expression trajectories is dependent on the quality of the GRN that is used. Therefore, we first assessed OneSC’s ability to infer GRNs using gold standard synthetic data and ground truth GRNs from the BEELINE platform (Pratapa *et al.* 2020) (Supplementary Info S3). We downloaded 10 sets of synthetic datasets (2000 cells, no drop-out) with gold standard GRNs, manually clustered the datasets using standard Scanpy (Wolf *et al.* 2018) pipeline, and used the provided Slingshot-computed pseudotime (Pratapa *et al.* 2020), which we provided as input to OneSC’s GRN inference function (Supplementary Figs S1–S3). Among the 10 gold standard GRNs, there are six synthetic networks: dyn-BF (12 edges and 5 nodes), dyn-BFC (18 edges and 9 nodes), dyn-CY (6 edges and 5 nodes), dyn-LI (8 edges and 7 nodes), dyn-LL (19 edges and 18 nodes), dyn-TF (20 edges and 7 nodes) and four literature curated networks: GSD (79 edges and 18 nodes), HSC (30 edges and 11 nodes), mCAD (14 edges and 5 nodes), and VSC (15 edges and 8 nodes). Then we computed precision and recall metrics of OneSC based on comparison of inferred GRNs to the ground truth GRNs (Supplementary Fig. S4A) and compared with 13 other GRN inference methods (GENIE3, Huynh-Thu *et al.* 2010; GRNBoost2, Moerman *et al.* 2019; PPCOR, Kim 2015; PyEpoch, Su *et al.* 2022a; LEAP, Specht and Li 2017; PIDC, Chan *et al.* 2017; SCRIBE, Qiu *et al.* 2020; SINCERITIES, Papili Gao *et al.* 2018; SINGE, Deshpande *et al.* 2022; SCODE, Matsumoto *et al.* 2017; GRISLI, Aubin-Frankowski and Vert 2020; GRNVBEM, Sanchez-Castillo *et al.* 2018; IQCELL, Heydari *et al.* 2022). Since OneSC produces a concrete network without edge weights like many other methods, to be as fair as possible in this comparison, we selected edge weight thresholds that optimized F1, the harmonic mean of precision and recall, for all the other methods that produce edge weights (all methods except IQCELL). OneSC achieved the highest mean F1 score at 0.61, followed by GRNBOOST2 with a mean F1 score of 0.49 (Fig. 2A). This result suggests that OneSC, without needing users to define thresholds post GRN inference, is comparable to other GRN inference methods when assessed on traditional GRN performance metrics (see also Supplementary Fig. S4A). In terms of runtime, OneSC is on average ranked as the second slowest method behind IQCELL (Supplementary Fig. S4B) despite that OneSC’s run time increases linearly with the number of genes in the network (Supplementary Fig. S4C).

Next, we assessed the robustness of OneSC to different parameter values (Supplementary Info S4). First, we explored how cluster assignment impacts GRN inference. We performed Leiden clustering on the benchmark data by finding the highest clustering resolution between 0.1 and 0.75 (0.05 increment) that still ensures there is at least one gene with substantial mean expression difference (>1.5) between all cluster pairs. This ensures that each cluster has a unique Boolean activity profile. To test the stability of the inference performance with when there are overclustering or under-clustering, we randomly split or merged clusters, performed network inference, and then determined the precision and recall. From this analysis, we found that there can be as large as a 50% decrease in F1 score when deviating from the optimal cluster assignment (Supplementary Fig. S5A). Therefore, we advise users to run OneSC with cell clusters that are meaningful and with distinct expression profiles given the biological context. Next, we tested the impact of varying the ideal number of edges for subnetworks (based on different subnetwork densities) and the number of GA generations but did not see noticeable differences in the performance when we adjusted user tunable parameters such as the ideal number of subnetwork edges (Supplementary Fig. S5B) or the number of generations for the GA optimization which is linearly correlated with runtime (Supplementary Fig. S6A and B).

Having determined the stability and performance of OneSC’s GRN inference approach, we next assessed the extent to which OneSC-inferred GRNs recapitulate the same number of steady states as in the gold standard data following the assessment used by the BEELINE benchmarking study (Pratapa *et al.* 2020). To perform this analysis, we ran 10 000 Boolean asynchronous simulations (following the Boolean function from the BEELINE benchmarking study; Pratapa *et al.* 2020) for each inferred GRN (given the same initial state that was used in BoolODE to generate the synthetic data) and then determined the number of distinct steady states reached. We found that OneSC generated the correct number of steady states in 5 out of 10 gold standard datasets with dyn-BFC, dyn-LL, dyn-LI networks generating one more steady state than gold standard networks, and HSC, mCAD networks generating two and one fewer steady states than gold standard networks, respectively (Fig. 2B). Next, we compared the similarity of inferred networks’ steady states to the gold standard steady states by calculating and finding the maximum percent agreement between the Boolean expression profiles from inferred networks’ steady states and those of the gold standard networks. The maximum percent agreement for each of the gold standard steady states in the synthetic datasets are averaged to show the overall similarity in terms of steady state profiles between inferred networks and gold standard networks (Fig. 2C). We found that steady states generated from OneSC networks were identical to the steady states of gold standard networks in dyn-BF (two steady states), dyn-BFC (one steady state), dyn-TF (three steady states), dyn-CY (zero steady state), and VSC (five steady states) structures (Fig. 2C). OneSC networks also generated steady states that were highly similar (average percentage of gene activity status agreement ≥0.86) to gold standard networks’ steady states in dyn-LI (one steady state) and dyn-LL (one steady state), GSD (two steady states) synthetic cell structures. We found overall lower average steady states similarity in OneSC’s HSC (four steady states) and mCAD (two steady states) inferred networks (Fig. 2C) likely due to the fact that these networks generated fewer steady states than gold standard networks (Fig. 2B). Overall, we found that networks generated using OneSC generally produced networks with the highest average steady state similarity (mean 0.87) followed by GENIE3 (mean 0.71) and second lowest divergent number of steady states (absolute mean 0.6) right underneath IQCELL (absolute mean 0.5). Taken together, this evaluation supports the notion that OneSC can capture enough of the key regulatory structure of the underlying GRNs to preserve dynamical properties such as the reachability and similarity of steady states.

### 3.2 OneSC’s simulation runtime scales linearly with the number of nodes in the network

The second central aspect of OneSC is the capability to generate single-cell expression states that reflect pseudotemporal trajectories using a Boolean network backbone. OneSC’s simulation platform is heavily inspired by a current method, BoolODE (Pratapa *et al.* 2020). Currently, BoolODE has two areas for improvement. The first is native support for simulating expression states upon network perturbations (i.e. knocking out or overexpressing genes). To address this, we have designed OneSC such that it is straightforward for users to test the impact of perturbations on pseudotemporal trajectories (see next section for example applications).

The second missing feature is computational scalability with the number of regulators per target gene. For example, it is infeasible to simulate expression trajectories with BoolODE for networks in which there are 10 or more regulators per target gene because the time complexity of the simulation grows exponentially with respect to the number of regulators (Dibaeinia and Sinha 2020). To reduce computational workload, we first eliminated the differential equations that model protein concentration dynamics. Second, we simplified regulatory functions using Boolean algebra such that the time complexity with respect to the number of regulators is linear.

**Figure 3.**
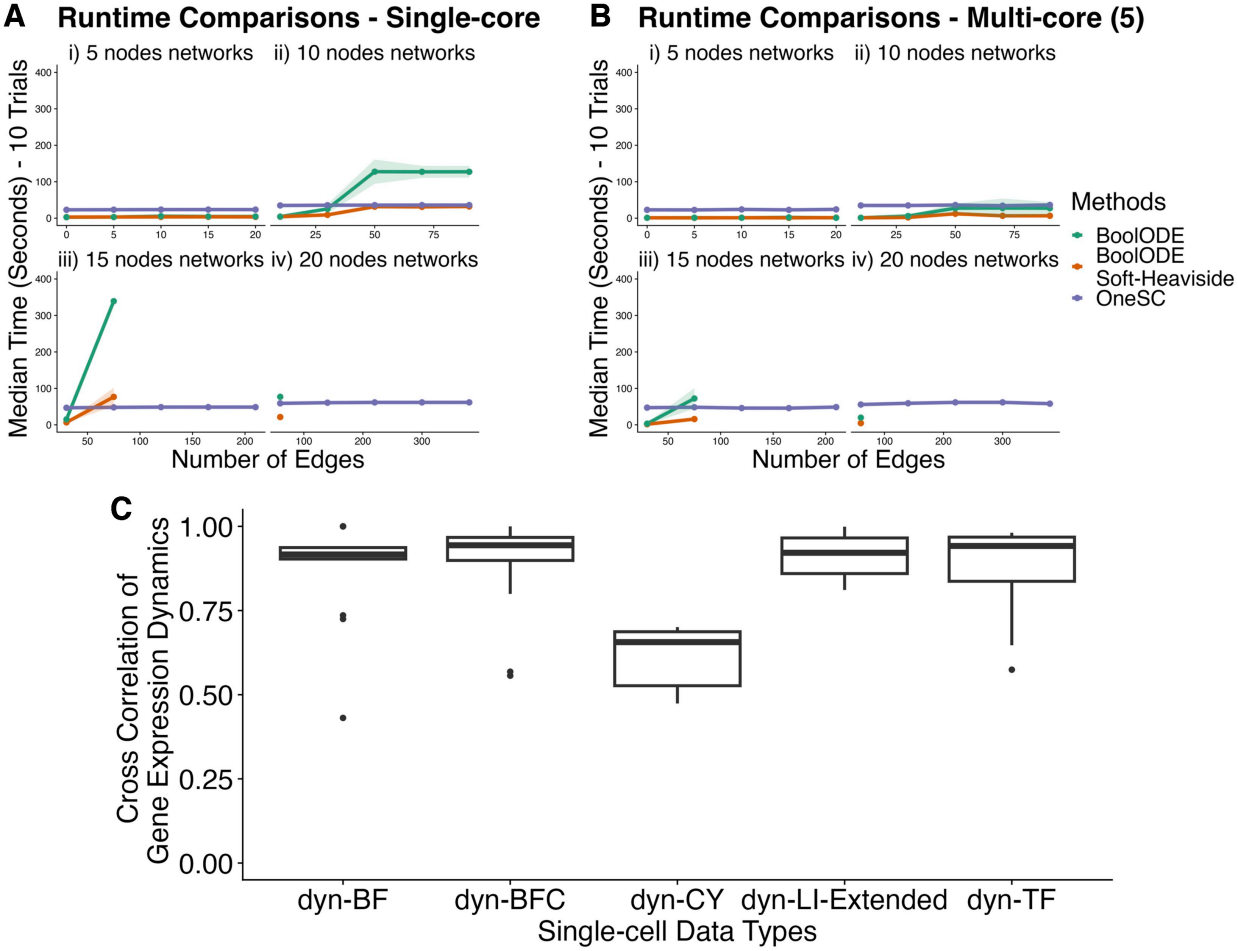
Runtime and similarity comparisons between OneSC simulator and BoolODE. (A) The median runtime for OneSC, BoolODE, and BoolODE (soft-heaviside) to simulate 10 random networks generated with different network sizes (i) 5, (ii) 10, (iii) 15, (iv) 20 and different network densities (0.2, 0.4, 0.6, 0.8, 1) without parallelization. The shaded area represents the range of runtime across 10 random networks simulations. If there is no data point for a particular network configuration, it means the simulation was not able to run. (B) The median runtime for OneSC, BoolODE, and BoolODE (soft-heaviside) to simulate 10 random networks generated with different network sizes (i) 5, (ii) 10, (iii) 15, (iv) 20 and different network densities (0.2, 0.4, 0.6, 0.8, 1) with parallelization (5 cores). (C) Cross-correlation of network gene expression dynamics between simulated data from OneSC and BoolODE. If the dataset has more than one trajectory, the cross-correlations of gene expressions are computed individually for each trajectory. To ensure the simulated data from BoolODE reached steady state, we resimulated dyn-LI gold standard network using BoolODE and extended the simulation time from 5 to 7.

We benchmarked OneSC simulator with BoolODE and BoolODE soft-heaviside (a faster version of BoolODE that also uses Boolean algebra) using 20 different unique network types across different network sizes {5, 10, 15, 20} and network densities {0.2, 0.4, 0.6, 0.8, 1} (Fig. 3A and B, Supplementary Info S5). There are 10 different random network structures for each unique network size and density configuration. For each random network, we ran five simulations with 5000 simulation steps turning off multicore parallel processing (Fig. 3A). With smaller networks (i.e. those with 5 and 10 nodes and fewer than 75 edges), simulations with BoolODE and BoolODE soft-heaviside completed more rapidly than those of OneSC (Fig. 3Ai, ii). However, at higher network size and density (i.e. those with 15 and 20 nodes and with more than 75 edges), OneSC simulations completed faster than those of BoolODE and BoolODE soft-heaviside (Fig. 3Aiii, iv). We found that BoolODE and BoolODE soft-heaviside failed to simulate at certain edge counts (120 edges with 15 nodes, 140 edges with 20 nodes) (Fig. 3Aiii, iv) as previously reported (Dibaeinia and Sinha 2020). Another observation to note is that while BoolODE’s runtime is sensitive to the number of edges and the number of nodes in the network, OneSC’s runtime is primarily correlated with the number of nodes and is less sensitive to the number of edges in the network. When using multicore parallelization to perform simulations, BoolODE and BoolODE soft-heaviside improved in terms of runtime for smaller networks: 5 node networks (Fig. 3Bi), 10 node networks (Fig. 3Bii), 15 nodes networks with less than 120 edges (Fig. 3Biii), 20 nodes network with less than 140 edges (Fig. 3Biv), while the runtime remained the same as single-core usage for OneSC. Consistent with single-core results, BoolODE and BoolODE soft-heaviside failed to simulate larger and denser networks (15 nodes network with more than 120 edges, 20 nodes network with more than 140 edges) (Fig. 3Biii, iv).

Next, we sought to determine the extent to which our reduction in the complexity of simulation equations and removal of protein simulation equations impacted the fidelity of OneSC’s simulations. To assess this, we compared OneSC’s simulated expression dynamics based on the gold standard GRNs to those generated by BoolODE, finding a high degree of cross-correlation between them (Fig. 3C). The dyn-CY (cyclic dynamical structure) simulation was the only exception and was likely due to differences in the periodicity of cyclic gene expression patterns. Furthermore, all the distinct clusters in the single-cell data generated by BoolODE are also observed from the single-cell data generated by OneSC (Supplementary Figs S7 and S8). Taken together, these results show that using OneSC for expression state trajectory simulations is more computationally tractable than other similar tools such as BoolODE.

### 3.3 Predicting the impact of TF perturbations on myelopoiesis

**Figure 4.**
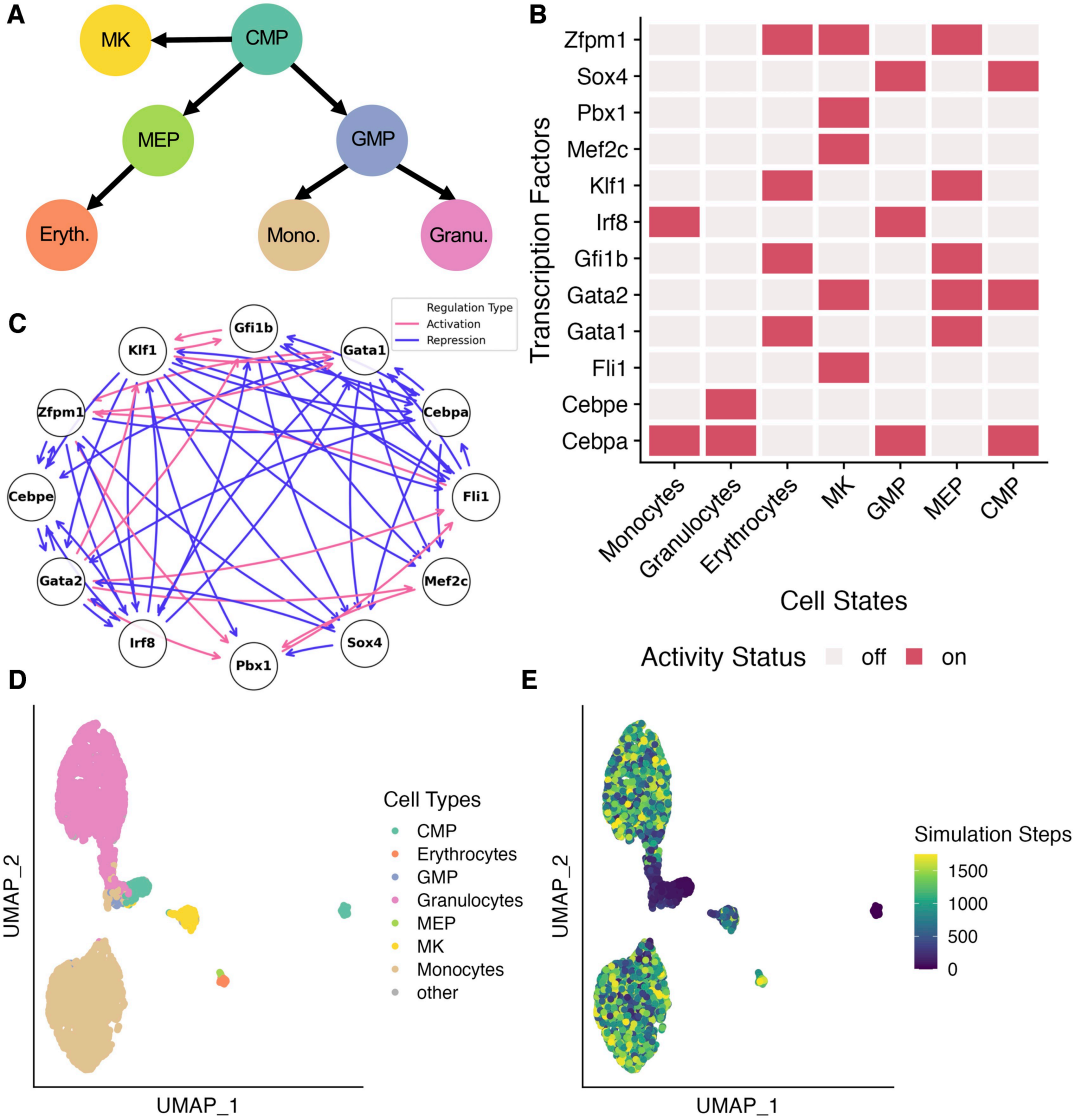
Application of OneSC to model developmental trajectories in mouse myeloid progenitor cells. (A) The cell state transition graph for mouse myeloid progenitor cells. (B) Heatmap representing the Boolean activity profiles of the 12 dynamically expressed transcription factors across seven cell states defined in the dataset. (C) OneSC’s inferred transcription factor circuit from mouse myeloid progenitor cells. Blue edges represent repression and red edges represent activation. (D) UMAP embeddings of synthetic cells across 200 simulation runs (sampled at every 50 time steps). The cells are labeled by the cell type with the lowest distance in Boolean activity profiles. Trajectories labeled as “other” are simulation runs in which the terminal state Boolean activity profile does not match perfectly with any of the Boolean profiles of the terminal cell types (MK, erythrocytes, granulocytes, monocytes). (E) UMAP embeddings of the synthetic cells across 200 simulation runs (sampled at every 50 time steps) colored by the simulation time steps.

To demonstrate the utility of OneSC on real single-cell expression data, we applied it to a scRNA-seq dataset of mouse myeloid progenitors (Paul *et al.* 2015) to infer a core TF circuit, to simulate differentiation trajectories, and to explore the consequences of perturbing these TFs on myelopoiesis. Prior to running OneSC, we clustered the cells and annotated as: common myeloid progenitor (CMP), erythrocytes, megakaryocytes (MK), monocytes, granulocytes, granulocyte monocyte progenitor (GMP), megakaryocyte erythrocyte progenitor (MEP) based on marker gene expression (Supplementary Table S1) (Supplementary Info S6) (Supplementary Fig. S9A). Then, we computed pseudotime using diffusion pseudotime (Haghverdi *et al.* 2016) and manually set the CMP cluster as the start of the trajectory and erythrocytes, MK, monocytes and granulocytes as the terminal cell states to construct the cell state transition graph (Fig. 4A). Under the classical hematopoiesis model, MK were thought to arise from MEP populations (Vannucchi *et al.* 2000, Klimchenko *et al.* 2009). In this dataset, MK have an earlier pseudotime than their nominal progenitor MEP (Supplementary Fig. S9A and B) consistent with recent recognition of an alternate direct differentiation from CMP to MK that bypasses an MEP intermediate stage in some contexts (Sanjuan-Pla *et al.* 2013, Notta *et al.* 2016, Miyawaki *et al.* 2017). Next, we used OneSC’s function to identify dynamically expressed TFs, which yielded 12 genes, all of which have been implicated in various aspects of hematopoiesis (Fig. 4B and Supplementary Fig. S9C). Finally, we used OneSC to infer a GRN (Fig. 4C), which we then used to simulate 200 simulation runs (i.e. synthetic single-cell trajectories) with 1800 simulation steps (analogous to pseudotime ordering), recapitulating the native cell state trajectories (Fig. 4D and E). The initial state was defined based on the activity status profile of the CMP cluster. Most of the 200 simulation runs stabilized into one of the four cell states (i.e. Boolean activity status profiles) that are identical to monocytes, granulocytes, MK or erythrocytes (Fig. 4D and Supplementary Fig. S10). Intermediate cell states such as MEP and GMP are occupied for brief intervals in some simulation runs, suggesting that the simulations follow developmental trajectories according to those in the training data (Supplementary Fig. S10). Looking at selected TFs expressions between simulated and real single-cell expression data (Supplementary Figs S11 and S12), we observed that simulated data recapitulates the selected marker genes of cell states found in the real data. For instance, both simulated cells and real monocytes cells express *Irf8*, an important TF for murine monocyte differentiation (Kurotaki *et al.* 2013), while granulocytic cells express *Cebpe*, a regulatory of granulopoiesis (Lekstrom-Himes 2001) (Fig. 4D and Supplementary Figs S9A, S11, and S12). On the other trajectory, both simulated and real erythrocytes express *Klf1*, an essential regulator for erythropoiesis (Siatecka and Bieker 2011) and MK express *Pbx1* (Fig. 4D and Supplementary Figs S9A, S11, and S12).

**Figure 5.**
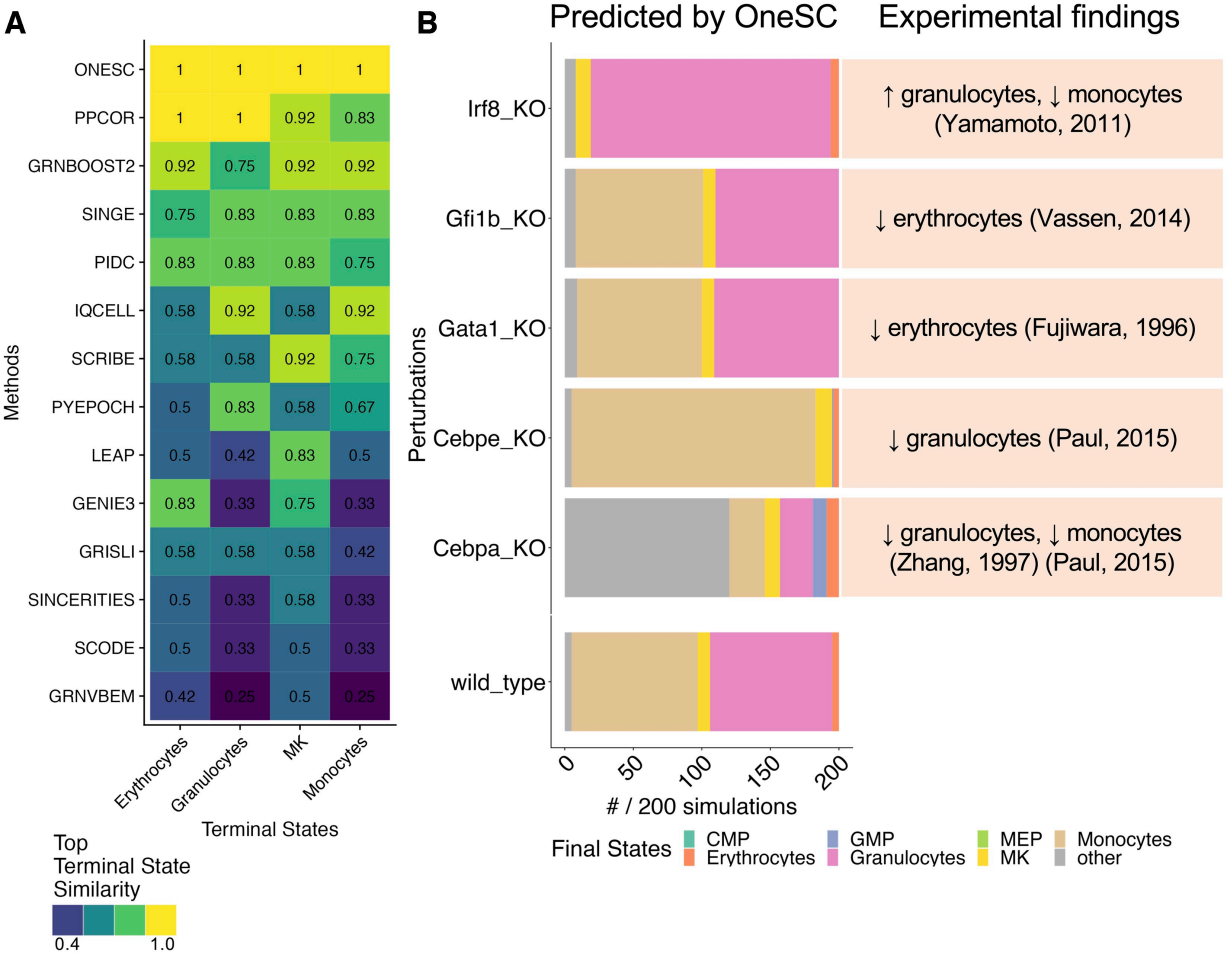
Assessment of OneSC network’s simulation fidelity and perturbation predictions. (A) Heatmap comparing the highest terminal state similarities of the networks generated by OneSC and other gene regulatory network inference methods for monocytes, MK, granulocytes, and erythrocytes. Similarity for each cell type is measured by highest percent agreement between Boolean activity profiles of the simulated terminal states and those from the real data. (B) Barplot showing the proportion of terminal states from OneSC’s knockout simulations of Irf8, Gfi1b, Gata1, Cebpe, Cebpa, and no perturbation simulation (wild_type) (left). The qualitative observations from previous published *in vivo* knockout experiments for Irf8, Gfi1b, Gata1, Cebpe, Cebpa (right).

Next, we explored the extent to which simulations with GRNs inferred by other methods mimic the myelopoiesis trajectories using OneSC’s simulator (Supplementary Info S7). Many of the GRN inference methods output an edge-weighted network instead of a concrete Boolean network. To convert those networks into concrete Boolean networks, we first identified the thresholds at which the resulting Boolean network would have the same number of edges as OneSC’s GRN or less if it exceeds the maximum possible number of edges. We found that OneSC network is the only network capable of reaching all the terminal states with 100% agreement in Boolean activity profiles when simulated (Fig. 5A). Accordingly, we found that the percentage overlap of regulatory edges between the concrete inferred networks of other methods and that of OneSC is correlated with the average similarity of simulated terminal states achieved by networks from other inference methods (Pearson correlation of 0.64) (Supplementary Fig. S13). This result highlights the fact that OneSC explicitly optimizes network configuration based on satisfying the meaningful cell states along differentiation trajectories inputted by the users while most GRN inference methods were designed to focus on edge recovery. In short, our results show that OneSC fills the need for inference of executable Boolean networks with dynamical properties that faithfully mimic cell type transitions in single-cell expression data.

Several of the TFs in our myelopoiesis GRN have been previously explored by generating transgenic mouse knockouts or by altering their expression. Therefore, we next explored how the biological consequences of these experimental perturbations related to perturbations that we could perform *in silico*. We simulated cellular trajectories upon knocking out and overexpressing each TF and recorded the final state composition (Fig. 5B and Supplementary Fig. S14). We examined five TFs for which we could find literature in which the TF was knocked out or perturbed in a relevant system.

First, we looked at the simulation of *Irf8* knockout. We found that there was an increase in granulocyte and decrease in monocyte final states (Fig. 5B). These results are largely consistent with experimental data. Mice lacking the *Irf8* gene (*Irf8*^−/−^ mice) have a disproportionate expansion of granulocytes/neutrophils at the expense of monocytes/macrophages (Scheller *et al.* 1999, Tsujimura *et al.* 2002, Yamamoto *et al.* 2011). In our simulation, *Irf8* overexpression influenced all the simulation runs to arrive at the monocyte state (Supplementary Fig. S14A) suggesting that *Irf8* is a key driver in the monocytic development (Tamura *et al.* 2000). When *Gfi1b* and *Gata1* were knocked out, there were fewer erythrocytes in the terminal states compared to those of wild-type simulations (Fig. 5B). This is in agreement with prior experiments in which *Gfi1b* knockout delays erythroid differentiation (Vassen *et al.* 2014). Similarly, *Gata1* knockout arrests development of red blood cell precursors (Fujiwara *et al.* 1996).

Our simulations of *Cebpe* knockout mirrored prior experiments in which loss of *Cebpe* arrests neutrophil progenitor development (Paul *et al.* 2015) (Fig. 5B). *Cebpa* knockout was previously found to arrest development of granulocytes and blocks granulocytes/monocytes initial specification leading to a loss of monocytes and granulocytes (basophils and neutrophils) (Zhang *et al.* 1997, Paul *et al.* 2015). *In silico Cebpa* knockout led to a drastic decrease in terminal state proportions of granulocytes and monocytes, and an increase in the number of GMPs and “other” cell types (i.e. terminal cell states with Boolean activity status profiles that do not completely match those of any of the real cell states) (Fig. 5B).

We have also tested other *in silico* perturbations without prior experimental verifications. For instance, when *Klf1*, an important TF regulating erythropoiesis (Orkin and Zon 2008, Tallack *et al.* 2010), was overexpressed *in silico*, the proportion of erythrocytes in terminal states increased (Supplementary Fig. S14A). On the other hand, when we knocked out *Klf1 in silico*, the proportion of erythrocytes in terminal states decreased (Supplementary Fig. S14B). Next we investigated the effect of perturbing *Zfpm1*, a required TF for the development megakaryocytic and erythrocytic lineage (Mancini *et al.* 2012). When *Zfpm1* was overexpressed, there were more final cell states that resemble MK or erythrocytes compared to those in wild-type simulations (Supplementary Fig. S14A). On the other hand, when *Zfpm1* was knocked out, there was a decrease of final cell states that resemble MK or erythrocytes (Supplementary Fig. S14B). Although majority of simulation results align with prior experimental results, there are also discrepancies between OneSC perturbation predictions and previous experimental findings. For example, prior findings show that Fli1 deficiency leads to a decrease in granulocytes populations in mice (Suzuki *et al.* 2013). However, OneSC simulations do not show noticeable decrease in granulocytes proportion when *Fli1* is knocked out (Supplementary Fig. S14B). Constitutive expression of Mef2c was previously shown to favor monocytic differentiation via inhibiting granulocytic differentiation (Schüler *et al.* 2008). However, OneSC overexpression simulations of *Mef2c* showed equal decrease in the proportion of monocytes and granulocytes terminal states and OneSC knockout simulations showed no change in the proportion of monocytes or granulocytes terminal states (Supplementary Fig. S14A and B). Taken together, these results at large show that OneSC can recapitulate the expression trajectories of mouse myeloid differentiation and can accurately predict the impact of genetic perturbations on terminal state composition.

## 4 Discussion

With the increase availability of scRNA-seq datasets of various biological systems, there has been a vast amount of interest in using these resources to infer the underlying transcriptional circuits and to develop a method to simulate the dynamics of these networks for predictions. Here, we present OneSC, an open-source Python package that can infer executable GRNs to faithfully recapitulate the developmental trajectories of biological systems and can stochastically simulate synthetic single-cell expression profiles that resemble single cells from real datasets.

We first benchmarked OneSC’s GRN inference capability using synthetic gold standard dataset from BEELINE platform (Pratapa *et al.* 2020) against other published GRN inference methods. Our results reveal that given the right clustering, OneSC can infer GRNs with precision, recall, and F1 scores that are comparable to the best-performing concrete networks achievable by existing GRN inference methods using an F1 maximization thresholding scheme. Different from most of the current GRN methods, OneSC does not require users to select a threshold post GRN inference to form a concrete network. This is an important feature because there is often no gold standard network for the user to identify the thresholds that maximize the F1 scores of inferred networks. Moreover, among the plethora of GRN inference methods, OneSC stands out for its capabilities to create concrete Boolean networks that recapitulate the terminal states observed in the training single-cell data, indicating that OneSC networks retain the essential regulations governing developmental processes. Secondly, OneSC’s simulator, heavily inspired by existing single-cell simulator BoolODE (Pratapa *et al.* 2020), overcomes the scalability issue of BoolODE (Dibaeinia and Sinha 2020) via the simplification of transcriptional regulation functions and the removal of protein production equations. OneSC has shown to be able to simulate denser and larger networks that BoolODE cannot simulate. Despite the simplification of equations for transcriptional regulations, OneSC can still generate different complex dynamical single-cell data types such as bifurcation or trifurcation. OneSC simulation platform also offers the flexibility for users to input their own GRNs. Lastly, we applied OneSC platform to real mouse myeloid single-cell dataset (Paul *et al.* 2015) and have demonstrated its capability of generating an executable GRN that captures the cell state transitions of different developmental trajectories. We have also demonstrated the predictive capabilities of OneSC by performing *in silico* perturbations of key TFs and saw that the changes in final cell state proportion largely match with experimental results in literature.

IQCELL is another integrative platform that performs Boolean network inference on single-cell data and simulates network with or without *in silico* perturbations (Heydari *et al.* 2022). IQCELL uses the Z3 engine to find the optimal logical rules, or regulatory functions, for each gene (Hamey *et al.* 2017) based on candidate interactions obtained from mutual information that best match single-cell data when executed as logical gates. With the inferred GRN, IQCELL uses asynchronous Boolean update to simulate under normal or perturbed condition (Heydari *et al.* 2022). Despite some similarities, there are several key differences between IQCELL and OneSC. First, OneSC uses an optimization method, GA, to identify the best set of regulatory interactions for each gene such that when simulated, the agreement between simulated and real activity status of target gene is maximized at cell type/cluster resolution instead of at single-cell resolution. Inferring GRNs at cell type/cluster resolutions via averaging the gene expression profiles of single cells in the clusters, although loses single-cell resolution, alleviates the issue of high noise due to drop-outs in scRNA-seq (Kharchenko *et al.* 2014, Hwang *et al.* 2018, Kim *et al.* 2020, Murphy and Skene 2022). Second, OneSC uses a continuous simulation system that allows users to simulate the continuous gene expression dynamics (including partial on and partial off), while IQCELL uses discrete asynchronous Boolean update that has two states (on and off). Lastly, IQCELL was only tested in linear developmental trajectories. The authors of IQCELL recommend users to infer a candidate GRN for each trajectory if there are multiple trajectories in the dataset (Heydari *et al.* 2022). This restriction limits the simulation and predictive capability of IQCELL when applied to single cell datasets with multiple trajectories and steady states. On the other hand, OneSC generates a Boolean network that encompasses multiple developmental trajectories allowing OneSC to simulate all the trajectories at once using stochastic differential equations and make perturbation predictions that model changes in cell fate decisions.

There are several limitations and caveats to our computational framework. First, as shown in our benchmarking results, the performance of OneSC network inference is heavily dependent on the clustering provided by the user. Many other GRN inference methods do not require users to input cell type/cluster information. To utilize OneSC to its maximum capabilities, we recommend users to cluster cells so that they represent distinct pseudotemporal cell states with distinct Boolean activity profiles of the network genes. To achieve this, users could identify the finest clustering resolution that still produces cell clusters with distinct expression profiles or perform subclustering to identify distinct cell states in coarse cell clusters (Luecken and Theis 2019). Second, while OneSC performs well at recovering structural edges from synthetic gold standard networks, OneSC is designed to infer regulatory interactions that are functional but not necessarily direct. Third, OneSC fundamentally infers Boolean networks to study cell state transitions during development and to predict *in silico* perturbations. Despite being an interpretable qualitative model of gene regulatory interactions that can generate predictive hypothesis via dynamical simulations (Wang *et al.* 2012, Barbuti *et al.* 2020), Boolean networks are limited by their discretization bottleneck (only on or off status) (Delgado and Gómez-Vela 2019). OneSC tries to mediate this issue via the adoption of BoolODE-like simulation scheme that allows partial activation or partial repression of regulators. However, we recognize that there are other methods to perform perturbation predictions focused on a GRN that are not constrained by Boolean output. For instance, there are regression-based methods such CellOracle (Kamimoto *et al.* 2023) and Dynamo (Qiu *et al.* 2022), and differential equation based methods such as SERGIO (Dibaeinia and Sinha 2020), PeTTSy (Domijan *et al.* 2016), RACIPE (Huang *et al.* 2018), and Dictys (Wang *et al.* 2023). There are also nonnetwork dependent generative modeling methods such PRECIENT (Yeo *et al.* 2021). Furthermore, with the increasing prevalence of perturb-seq data (Dixit *et al.* 2016), there are various new machine learning and deep learning methods (Lopez *et al.* 2022, Roohani *et al.* 2024) that train models from perturbation data for predicting genetic perturbations (benchmarked in Kernfeld *et al.* 2024). Fourth, the input cell state transition graph must be hierarchal and non-cyclic with an initial state and at least one terminal state. The later cell states on the developmental hierarchy cannot have a transitional edge to an earlier cell state on the developmental hierarchy. In the case of cyclic transition graphic structure, we recommend the users to break it into linear structure before using as input in OneSC. Fifth, even though OneSC is capable of simulating perturbations of multiple genes simultaneously, we have only assessed and validated the results of a handful of single TF perturbations here. Users should take caution when interpreting results from perturbation simulations with multiple genes. Sixth, although we have developed simple helper functions to automatically construct the cell state transition graph based on pseudotime ordering and cluster expression similarity, we recognize that these functions may not perform well for all biological systems. Therefore, when the outputted state transition graph is incorrect, users should manually create the cell state transition graph with the help from specialized tools such as partition-based graph abstraction (PAGA) (Wolf *et al.* 2019), RNA velocity tools (La Manno *et al.* 2018, Bergen *et al.* 2020), CellRank (Lange *et al.* 2022). This also highlights the urgent need for automated tools to accurately infer cell state transition graphs from single-cell datasets. Seventh, steady state reachability depends on the set of TFs in the GRN, and therefore this set may need to be expanded or reduced if simulations do not reflect real steady states. The selected set of TFs must show sufficient level of variabilities between cell state clusters. Lastly, with the emergence of single-cell multiomics (Lee *et al.* 2020, Dimitriu *et al.* 2022), there has been an explosion of GRN inference methods that utilize data modalities such as chromatin accessibility beyond gene expressions (Badia-I-Mompel *et al.* 2023). Several of the current methods use ATAC-seq data to construct a base network by identifying TF motifs in the accessible promoter or enhancer regions (Badia-I-Mompel *et al.* 2023). For future work, we can adapt similar techniques of integrating ATAC-seq to identify potential regulators and use those as constraints to fine-tune the inferred networks.

## 5 Conclusion

In conclusion, we present a computational tool, OneSC, that performs two main tasks. First, it infers executable gene regulatory Boolean networks from single-cell expression profiles, inferred pseudotime and cluster/cell type annotation given by the users. Second, OneSC performs dynamic simulations that generate synthetic single cells to mimic trajectories and cell state transitions. Coupling these two main functions, users can perform *in silico* gene perturbations to predict shifts in terminal cell states, blockage or promotion of certain lineages and changes in gene expression dynamics. To promote accessibility of OneSC (https://github.com/CahanLab/oneSC), we have made the code freely available under an Open-Source license and as an easily installable Python package.

## Supplementary Material

btae703_Supplementary_Data

## Data Availability

OneSC is available as a Python package on Github (https://github.com/CahanLab/oneSC) and Zenodo (https://zenodo.org/records/14052421). Code to reproduce the analyses and results of this study is available on GitHub (https://github.com/CahanLab/onesc_paper).
