# Supplementary material for: OneSC: a computational platform for recapitulating cell state transitions
Source: Bioinformatics. 2024 Nov 21;40(12):btae703. doi: 10.1093/bioinformatics/btae703 (PMC11630913; doi:10.1093/bioinformatics/btae703)
Supplement: btae703_Supplementary_Data [file btae703_supplementary_data.zip › Supplementary_material[AU].pdf]

Supplementary Material for OneSC: A computational platform for recapitulating cell state transition

Table of Contents

Supplementary Information 2

1. *OneSC – Network inference method* 2

 1A. Construction of cluster transition graph 2

 1B. Identification of dynamically expressed transcription factors 4

 1C. Curation of cluster/cell type Boolean expression profiles 4

 1D. Inference of executable GRNs using genetic algorithm 7

2. *OneSC – Simulation method* 11

3. *Benchmarking GRN inference methods* 13

4. *Parameter sensitivity analysis for OneSC* 14

5. *Comparisons of OneSC simulator and BoolODE* 15

6. *Processing of mouse myeloid progenitors single-cell data* 16

7. *Benchmarking inferred myeloid differentiation networks* 16

8. *Assign cell type annotations to simulated cells* 17

Supplementary Figures 18

Supplementary Table 36

References 37

Supplementary Information

1. OneSC – Network inference method

The workflow of OneSC GRN inference is divided into four main sections A) construction of cell cluster transition graph, B) identification of the most dynamically expressed transcription factors (TFs), C) curation of cluster/cell type level Boolean expression profiles for regulatory network inference and D) network inference using genetic algorithm. Step 1 and 2 can be skipped if the dynamically expressed TFs or cluster transition graph are defined independently.

1A. Construction of cluster transition graph

The initial step in the OneSC inference process involves creating a graph that illustrates the transition between different cell type clusters along cell state transition trajectories. This method is heuristic and may not be applicable to all biological systems. Users can also manually construct the cell state transition graph based prior biological knowledge or modify the output graph from OneSC's helper function.

OneSC first calculates both the average expression profiles and the average pseudotime for each cell type cluster. Then OneSC determines all feasible transitions from a "parent" cluster to a "child" cluster, with the condition that the average pseudotime of the parent cluster must be less than that of its children. For each possible cell cluster transition pairs, a connectivity score is computed using the scaled Euclidean distance of the expression profiles and average pseudotime of parent cell cluster.

$$connectivity\ score_{AB} = (scaled\ dist_{AB}) + (\frac{pseudotime_A}{2})$$

$$scaled\ dist_{AB} = \frac{dist_{AB} - dist_{min}}{dist_{max} - dist_{min}}$$

$dist_{max}$ represents the largest distance between all possible cluster pairs and $dist_{min}$ represents the smallest distance between all possible cluster pairs. The scaling process confines the scaled distance between 0 and 1.

The connectivity score is based on the idea that if cell type A (parent) is directly transitioning into cell type B (child), their expression profiles should be similar (i.e have small distance), as there should be small changes between two adjacent clusters in the trajectory. Additionally, the pseudotime of cell type A (the parent) should be small, indicating that it is significantly upstream

of its child cluster. By combining pseudotime of parent cluster with finding the smallest expression profiles distance, this approach allows OneSC to find parent-child cluster pairs in which the expression profiles are similar, but the parent cluster is substantially upstream of the child. It is important to note that connectivity $score_{AB}$ does not necessarily equal connectivity $score_{BA}$ because the term that uses the pseudotime of the parent cell cluster will be different.

During the construction of cell state transition graph, OneSC adds incoming and outgoing edges independently for each cell type cluster. Duplicated edges (same nodes and same direction) are removed at the end. For the cell type clusters that are not defined as initial or terminal (assigned by user), OneSC constructs one incoming edge and one outgoing edge by selecting the feasible transition pairs with the lowest connectivity scores. For clusters that are defined as initial or terminal, OneSC finds one incoming edge or one outgoing edge respectively by selecting the feasible transition pair with the lowest connectivity score.

Using this method, it is possible that two or more clusters can have incoming edges from the same parent cell cluster (branching) or a cell cluster can have incoming edges from two or more parent cell clusters (converging).

Here is a possible example of creating a branching transition graph (multiple children) that also fulfills the criteria listed above. We have nodes A (initial), B, C (terminal), D (terminal), and during the transition graph construction

node A gets 1 outgoing edge to node B

node B gets 1 incoming edge from node A and 1 outgoing edge to node C

node C gets 1 incoming edge from node B

node D gets 1 incoming edge from node B

If we compile the network and remove duplicates, we will get

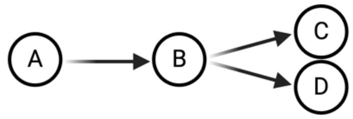

Here is a possible example of creating a converging transition graph (multiple parent) using the criteria listed above. We have nodes A (initial), B, C, D (terminal), and during the transition graph construction

node A gets 1 outgoing edge to node B

node B gets 1 incoming edge from node A and 1 outgoing edge to node D

node C gets 1 incoming edge from node A and 1 outgoing edge to node D

node D gets 1 incoming edge from node B

If we compile the network and remove duplicates, we will get

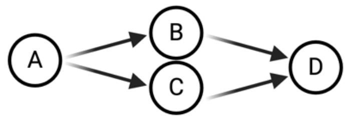

If the cell state transitions have been previously characterized, the users are free to manually curate the cell state transition graph using the networkx package.

1B. Identification of dynamically expressed transcription factors

Using user inputted cluster/cell type annotation, pseudotime ordering and cell state transition graph, OneSC identifies the most dynamically expressed transcription factors (TFs) by fitting a generalized additive model (GAM) to predict individual gene expression using pseudotime across a trajectory (Su *et al.*, 2022; Trapnell *et al.*, 2014). TFs are considered dynamically expressed if they are sufficiently expressed in at least one cell cluster (default percent expression of 0.1), have an adjusted p-value for the GAM smooth term for pseudotime below the defined threshold (default 0.05) and a maximum log-fold change between all cluster pairs greater than the defined threshold (default 2). Adjusted p-value <0.05 for GAM smooth term suggests there is a statistically significant relationship between gene expression and pseudotime. In the case with multiple trajectories, OneSC would fit GAM models individually for each trajectory and gather all the TFs that are considered to be dynamically expressed in at least one trajectory. Users can also manually curate the set of core TFs based on prior knowledge provided that the selected TFs must be sufficiently expressed in at least one cell state cluster and must show sufficient level of expression variability between cell state clusters such that they are turned off in at least one cell state cluster and turned on in at least one cell state cluster.

1C. Curation of cluster/cell type Boolean expression profiles

Different from many scRNA-seq based GRN inference method, OneSC infers GRN based on the binarized cluster/cell type activity profiles rather than the expression profiles of individual

cells. For each gene, OneSC curates the activity profiles (on/off) of itself as target gene labels and the activity profiles for all the transcription factors (TFs) in the network as the regulator activity profiles across different cell states in different trajectories. The goal is to generate a subnetwork of regulators that regulate the target gene such that the simulated Boolean output of the target gene generated from the activity profiles of the regulators and the subnetwork configuration is in agreement with the target gene's observed activity profiles across all cell states. This section mainly focuses on the curation of target gene labels and regulator activity profiles for the network genes as input for genetic algorithm optimization. The details of the network inference and optimization process will be discussed in the next section.

OneSC first identifies the expression thresholds that binarize the cluster/cell type level gene expression profiles into 1 or 0 (on or off) for each gene. OneSC first averages the expression profiles of single cells for each cell state cluster. Based on the highest cluster-averaged expression ($\text{clusterExp}_{\text{max}}$) and the lowest cluster-averaged expression ($\text{clusterExp}_{\text{min}}$), OneSC selects the threshold for different genes using

$$thresh = (clusterExp_{max} - cluster\text{Exp}_{min})(percent_{cutoff}) + cluster\text{Exp}_{min}$$

$percent_{cutoff}$ has a default value of 0.4, which can be adjusted by the users. A gene is considered to be turned on (activity status of 1) in a cluster if the averaged gene expression is greater than the expression threshold and the cluster has at least 30 percent of the cells expressing the gene (user tunable parameter). Using the expression thresholds, OneSC generates the activity profiles of all genes across different cell states.

Next OneSC curates the activity status labels for each gene and its accompanying regulator activity profile across all the cell states. This process is done on a trajectory by trajectory basis (i.e the activity status for a target gene and its accompanying regulator activity profiles across cell states are curated independently for individual trajectories). The regulator activity profiles and the activity status labels of the target gene from different trajectories are concentrated together before inputting to genetic algorithm for subnetwork optimization that maximizes the agreement of target gene status labels across all cell states. For example, in the toy branching cell state transition graph (see below), there are two trajectories: trajectory 1 is composed of clusters A, B, C and trajectory 2 is composed of clusters A, B, D

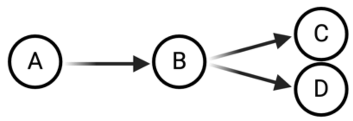

OneSC independently curates the activity status and regulator activity profiles of a target gene in the cell states associated with trajectory 1 and trajectory 2. It then combines the results, resulting in the curation of target gene activity status and regulator activity profiles for 6 cell states: A1, B1, C1 from trajectory 1 and A2, B2, D2 from trajectory 2. Lastly, cell states with the conflict of having same regulator activity profiles but different target gene status are removed as there are no possible subnetwork solutions.

For each individual trajectory, OneSC also identifies and records the pseudotime points at which genes change activity status (going above or below the expression threshold). OneSC first averages the single cell expression profiles within a pseudotime bin (default value of the pseudotime bin is set as 0.01) across the trajectory and then finds the pseudotime points at which the genes change status. This helps OneSC identify the precise order in which TF regulators or target gene change status during cell state transitions.

Here are the details on how to curate target gene status and regulator activity profile for the cell states in a trajectory. If target gene does not change status during transition of current cell state to the next, that means the regulator activity profile at the current cell state does not promote transition of the target gene (stable state). OneSC would then assign the target gene activity status and the regulator activity profile based on the Boolean activity status of the target gene and TF regulators at the current cell state. If the target gene activity status changes during the transition from the current cell state to the next, that means the regulator activity profile at the current cell state promotes transition of the target gene (unstable or transitional state). In this case, OneSC would use the activity status of the target gene in the next cell state (post transition) and the regulator activity profile with the activity status of all the TF regulators before the regulatory window of the target gene transition (default value of the regulatory window is set to be 0.01 pseudotime units prior to the target gene's activity change).

To better characterize the regulator activity profile right before the transition of the target gene, OneSC uses the precise pseudotime points in which genes are turned on or turned off to refine the inference of causal relationship between genes. Consider a scenario where the activity status of a target gene changes during transition from cell state N into cell state N+1 and transcription factor A also changes activity status, but after the regulatory window. OneSC would use the activity status of transcription factor A at cell state N in the regulator activity profile because the change in transcription factor A occurs after the target gene's regulatory window, indicating that transcription factor A's new status do not contribute to the transition of target gene.

On the other hand, if target gene's activity changes during transition from cell state N into cell state N+1 but transcription factor A changes activity at an earlier point than target gene (before the regulatory window), then OneSC will use the activity status of transcription factor A at cell state N+1 in the regulator activity profile because transcription factor A's new status at cell state N+1 could influence the transition of the target gene.

To further use pseudotime to finetune the causal relationship between genes, OneSC categorizes TFs that are first turned on at a cell state later than target gene in a trajectory as 'unlikely activator'. This is under the assumption that an activator should be turned on earlier than the target gene. During the network inference and optimization step, there will be a penalty term to discourage transcription factor A from forming activating edge with target gene. However, it is possible to still infer transcription factor A as an activator if the activating edge increases the agreement between Boolean simulated status and observed target gene status. The specific details will be described in the next section.

OneSC repeats this data curation of target gene activity status and regulator activity profiles for all the genes in the network. If target gene is a transcription factor, it will also be a part of the regulator activity profiles to potentially act as a self-activator. If the target gene is turned on at cell state N, then the activity status of target gene in the regulator activity profiles for all the downstream cell states after cell state N are encoded as 1 (on). This is under the assumption that if the target gene can self-activate, once it is turned on it will be able to sustain itself.

1D. Inference of executable GRNs using genetic algorithm

With the target gene activity status labels and the regulator activity profiles of transcription factors for each gene, OneSC next optimizes the potential regulatory interactions (activation: 1, repression: -1, or no regulation: 0) between different regulators and target gene. OneSC uses a repressor centric scheme such that if there is at least one repressor active (activity status of 1), then the target gene activity is simulated to be inactive (activity status of 0). The Boolean rule of activators and repressors is written as

$$(A_1 \vee A_2 \vee A_3 \vee \dots) \wedge \neg R_1 \wedge \neg R_2 \wedge \neg R_3 \wedge \dots$$

If at least one activator is active and no repressor is active then the target gene activity is active. If at least one activator is active and at least one repressor is active, then the target gene activity is inactive. If all activators are inactive then the target gene activity is inactive.

If there are no activators presented in regulatory network of target gene, then the Boolean rule is

$$\neg R_1 \wedge \neg R_2 \wedge \neg R_3 \wedge \dots$$

If all repressors are inactive then the target gene activity is active. If at least one repressor is active then the target gene activity is inactive.

If there are no repressors presented in the regulatory network of target gene, then the Boolean rule is

$$A_1 \vee A_2 \vee A_3 \vee \dots$$

If at least one activator is active then the target gene activity is active. If all activators are inactive then the target gene activity is inactive.

To perform optimization, OneSC first identifies the most optimal subnetworks for each gene and then combines all the subnetworks into a larger GRN. The target gene subnetwork is composed of regulators that have a direct edge towards the target gene. The subnetwork could be represented as a regulatory vector that represents the regulatory relationships of the regulators on the target gene with the length equal to the number of possible regulators.

$$\begin{bmatrix} v_1 \\ v_2 \\ v_3 \\ \dots \\ v_{nreg} \end{bmatrix}$$

Each v_i can be encoded in 3 ways $\{-1, 0, 1\}$ representing repression, no regulation and activation respectively for the target gene. Guided by the target gene activity status label and regulator profiles across different cell states, OneSC uses genetic algorithm (Albadr *et al.*, 2020; Katoch *et al.*, 2021) to identify the regulatory relationship vector that maximizes the agreement between activity status of the target gene outputted by the Boolean simulation (based on the activity status of assigned activators and repressors) and the activity status of target gene observed across different cell states in the real data.

Genetic algorithm (GA) is a metaheuristic optimization process inspired by natural selection. In short, genetic algorithm first starts with an initial population of random subnetworks for a target gene. Each subnetwork is evaluated using a fitness function that favors agreement of the activity status of the target gene outputted by the Boolean simulation and the activity status of target gene observed in the real data across different cell states. Then the set of subnetworks

with high fitness are selected as parents and are subjected to crossover (random shuffling between subnetworks) and mutation (random changes to subnetworks) to generate next generation of subnetworks population. The process is repeated until it converges to an optimal fitness or reach the maximum generation limit. To overcome the stochastic nature of genetic algorithm and ensure robust outputs, the process of subnetwork optimization is repeated multiple times with different initial populations and GA evolution processes for genetic algorithm (default 25 times with 5 different seeds for generating different initial populations and 5 different seeds for GA's evolution processes). In the ensemble of inferred subnetworks, the majority subnetwork structure is voted to be optimal stable subnetwork. If there is a tie for the majority subnetwork, the subnetwork that has the number of edges closest to the ideal number of subnetwork edges (set by the users) wins. OneSC uses PyGAD (Gad, 2021) (3.1.0) to perform genetic algorithm optimization.

The fitness function for the genetic algorithm to maximize is made of several components that help achieve maximum target gene activity status agreement and also satisfy other secondary constraints. It is split into reward components (the higher the better) and penalty components (the lower the better).

$$\begin{aligned} fitness = {} & target\ gene\ correctness\ reward + additional\ edge\ reward \\ & + regulatory\ direction\ agreement\ reward + similar\ stage\ reward \\ & - self\ inhibition\ penalty - no\ edge\ penalty - unlikely\ regulator\ penalty \\ & - auto\ regulation\ penalty\ (can\ be\ turned\ off) \end{aligned}$$

$target\ gene\ correctness\ reward$ represents the reward given to the number of agreements between the Boolean simulated activity status and the activity status observed across cell states for the target gene. It is defined as

$$(\#\ of\ target\ gene\ activity\ status\ agreements)(10^4 \times n_{regulators})$$

The rationale for adding $n_{regulators}$ is to ensure that the reward scales with the number of genes in the network. The large constant (10^4) ensures that the primary focus of the fitness function is to achieve of the highest number of agreements for target gene activity status across cell states.

$additional\ edge\ reward$ represents the reward given to every new functional edge (activation or repression) added between regulators and target gene. The goal is to promote adding new regulatory edges every new generation (if there are no other penalties). The reward is 10^2 for each repressing or activating edge. However, if the number of regulatory edges surpasses the

ideal number of regulatory edges (user defined parameter) for the target gene, then this term becomes -10^2 to encourage the removal of unnecessary edges. It is worth noting that in our benchmark (**Supplementary Fig S5B**), we found that setting the ideal subnetwork density as 0.3-0.4 seems to yield the highest performance. The conversion between ideal number of regulatory edges for the target gene and subnetwork density is

$$ideal\ \#\ of\ edges = subnetwork\ density\ \times\ \#\ of\ regulators$$

$regulatory\ direction\ agreement\ reward$ represents the reward given for every activating or repressing edge between regulators and target gene with the direction of regulation that matches the direction of correlation between regulators and target gene expression. If the direction matches, then the reward is $10 \times Pearson\ correlation$. Otherwise, the reward is $-10 \times Pearson\ correlation$. The rationale of this term is to favor regulatory interactions that match correlation patterns.

$similar\ stage\ reward$ represents the reward given to regulatory edges where regulators are first active at a similar stage on the cell state transition hierarchy as the target gene. The assumption is that if a regulator is turned on around the similar stage as the target gene (one stage earlier or same stage), then it is more likely to be an activator or a mutual inhibitor (during branching cell fate decisions). All the regulators are assigned a weight that is inversely related to the cell state at which it is first active (i.e terminal state in the longest trajectory would get a weight of 1 and initial state would get a weight equivalent to the maximum number cell states along the longest trajectory). If regulator's weight is less or equal to target gene's weight + 1, then the reward would be $+\ regulator\ weight$ for an activating or repressing edge. This allows the algorithm to prioritize regulators that are first active ideally one cell state earlier than the target gene or the same cell stage on the cell state transition hierarchy as the target gene. Otherwise, this term would be -1 to penalize regulatory edges with regulators that are first turned on much earlier than the target gene.

$self\ inhibition\ penalty$ represents the penalty to prevent the formation of self-inhibitory edge. The penalty is $3 \times 10^4 \times n_{regulators}$ which is 3 times the reward for matching the activity status of the target gene for a cell state. The rationale is to prohibit all potential auto-inhibitory edges. There are instances of self-repression in transcription factors such as Hes family (Chen *et al.*, 2005; Bessho, 2003) resulting in oscillation in the expression dynamics. But there are insufficient amount of information from mRNA expression profiles to properly infer this

relationship, and it typically requires additional sets of differential equations for protein dynamics to properly model the oscillation (Momiji and Monk, 2008).

$no\ edge\ penalty$ represents the penalty to prevent the situation of no regulatory edges for a target gene. The penalty is $3 \times 10^4 \times n_{regulators}$ which is 3 times the reward for matching the activity status of the target gene for a cell state. The rationale is to remove the possibility of having genes in the network that do not interact with any other genes or itself.

$unlikely\ regulator\ penalty$ represents the penalty for adding regulatory edges with regulators that are previously defined as unlikely to activate the target gene during the training data curation step. The penalty is 10^2 per regulatory edge with an unlikely regulator. By default, the unlikely activators are genes that are first active at a later cell state than the target gene. This penalty works in conjunction with $similar\ stage\ reward$ to help pinpoint the potential activators that are first active one cell state before the target gene activation. OneSC also allows users to manually add unlikely activators or unlikely repressors for each target gene based on prior information.

$auto\ regulation\ penalty$ represents the penalty for self-activating edge (can be turned off by the user). This is to remove unnecessary self-activating edges. The penalty is 2×10^2.

2. OneSC – Simulation method

OneSC, like BoolODE (Pratapa *et al.*, 2020), has a stochastic differential equation for each gene to model the dynamics of that gene's expression.

$$\frac{d[x_i]}{dt} = mf(Ri) - l_x[x_i] + s\sqrt{[x_i]}\,\Delta W_t$$

$$\Delta W_t = \mathcal{N}(0, h)$$

$[x_i]$ represents the expression level of target gene, $f(Ri)$ represents the regulatory function capturing all the regulators and their interactions with target gene, m is the transcription rate (default value is 0.2), l_x is the degradation rate (default value 0.1), s is the strength of the noise (default value is 2), ΔW_t is the change in Wiener process increment sampled from a normal random distribution with mean zero and variance equivalent of simulation time step h (default value is 0.01). OneSC use the Euler-Maruyama method (Kloeden and Platen, 1992) to numerically integrate the stochastic differential equations.

$$[x_i] = x_{i-1} + [mf(R_{i-1}) - l_x[x_{i-1}]](h) + s\sqrt{[x_{i-1}]}\,\Delta W_t$$

To perform in-silico perturbation, a perturbation term D is added to the equation.

$$[x_i] = x_{i-1} + \left[(m + D)f(R_{i-1}) - l_x[x_{i-1}]\right](h) + s\sqrt{[x_{i-1}]}\,\Delta W_t$$

To simulate overexpression, assign D to be a positive number (1 by default). To simulate knockout, assign D to be a negative number (-1 by default). The range of expression for target gene during simulation is constrained to be between 0.02 and 2 by default (user tunable) if the expression values exceed this range.

We made two strategic decisions when implementing OneSC to enhance its computational efficiency. First, OneSC does not have equations to model protein dynamics. Second, instead of modelling all 2^Ncombinations of TF states, OneSC employs Boolean algebra (Shannon, 1938) to model the transcription regulation. The algebraic forms of various Boolean logic are listed in Table 1.

Boolean Operators	Boolean Algebra
P_1 or P_2	1 – (1-P_1)(1-P_2)
P_1 and P_2	(P_1)(P_2)
not P_1	1 – P_1

Table 1. Boolean Operators and their corresponding Boolean algebraic form.

It is worth noting that similar approach was also used in the soft-heaviside version of BoolODE (Jalihal, 2020). P in Table 1 represents the activity probability of a transcription factor from 0 to 1. The activity probability can be transformed from the expression values via a logistic function.

$$P_{TF} = \frac{1}{(1 + e^{-a([TF]-b)})}$$

a adjusts for the steepness of the logistic function (default value is 7). $[TF]$ represents the expression values of the transcription factor. b represents the mid-point between max and min expression values. Consider the example shown in (Pratapa *et al.*, 2020), if a gene (denoted by X) has two activators (P, Q) and one inhibitor (R)

$$X = (P \vee Q) \wedge \neg(R)$$

Combining Boolean polynomials, the regulatory function would be written as

$$f(Ri) = \left(1 - (1 - P_p)(1 - P_Q)\right)(1 - P_R)$$

3. Benchmarking GRN inference methods

Synthetic data generated from BoolODE (Pratapa *et al.*, 2020) (network structures include LI, CY, LL, BF, BFC, TF, mCAD, VSC, HSC, GSD) were downloaded from https://doi.org/10.5281/zenodo.3378975 (version 3). For each synthetic gold standard network, the dataset (sample id: 1) with 2000 cells and no dropout was used to benchmark OneSC against other methods. Clustering of the synthetic data was performed with Scanpy (1.9.1) (Wolf *et al.*, 2018) prior to running OneSC pipeline. 10 neighbors (n_neighbors=10) and 9 PCs (n_pcs=9) were used to construct the neighborhood graph (sc.pp.neighbors). The Leiden clustering resolution for the synthetic data were LI: 0.25 CY: 0.45, LL: 0.75, BF: 0.55, BFC: 0.55, TF: 0.3, mCAD: 0.15, VSC: 0.25, HSC: 0.25, GSD: 0.75. These clustering resolutions were identified by finding the highest resolution between 0.1 and 0.75 (0.05 increment) that still ensures there is at least one gene with substantial mean expression difference (>1.5) between all cluster pairs. In the dyn-TF synthetic dataset, two clusters with similar expression states were combined forming now labelled cluster 4 (**Supplementary Fig S2B**). In the HSC synthetic dataset, two extremely small clusters (with fewer than 20 cells) were removed. All the benchmarks were performed on AWS c5.4xlarge EC2 instance running Ubuntu. IQCELL was executed using default parameters shown in its tutorial. For datasets with multiple trajectories, trajectory specific GRN was inferred using IQCELL as suggested by its authors.

To convert the edge weighted network into concrete networks for fair comparisons, a thresholding scheme was used to generate concrete networks that maximizes the F1 score. The precision, recall and F1 scores were calculated for each edge weight threshold and the edge weight threshold that produced the highest F1 score was chosen. All the edges with edge weights that were larger or equal to the threshold were kept as part of the concrete network. For a regulatory edge to be considered a true positive, the edge must have the correct regulator, target gene and type (activating or repressing). For methods that do not generate the type of regulation (GENIE3, GRISLI, GRNBOOST2, LEAP, PIDC, SCRIBE, SINGE), the sign of the partial Pearson correlation between regulator expression and target gene expression was used to assign the type of regulation.

Asynchronous Boolean update from BoolNet R package (2.1.9) (Müssel *et al.*, 2010) was used to simulate the steady states of inferred concrete networks (with the exception of IQCELL). 10,000 asynchronous Boolean simulations, with the initial states provided on BoolODE Github (https://github.com/Murali-group/BoolODE/tree/master/data), were ran to identify the possible steady states for inferred networks. The default simulation platform from IQCELL was used to

simulate its trajectory specific networks. Next we quantified the agreement in Boolean status (1 or 0) of two steady states using the following equation.

$$percent\ agreement = \frac{\#\ of\ genes\ that\ match\ status}{total\ \#\ of\ genes}$$

For each steady state generated by the gold standard network, a list of percent agreements was calculated between the gold standard steady state and all the steady states generated by the inferred network. The maximum percent agreement was selected for each steady state in the gold standard network and averaged by the total number of steady states in the gold standard network to determine the average steady state similarity between inferred networks and gold standard network.

4. Parameter sensitivity analysis for OneSC

To explore the impact of over- and under-clustering, we randomly selected a cluster to either split into two (for over-clustering) or merge with previous cluster (for under-clustering). In the case of testing under-clustering, the selected cluster was merged with its parent as defined by the cluster transition graph. When testing over-clustering, the random cluster was split in two such that cells with pseudotime < cluster-averaged pseudotime formed one cluster and all other cells are portioned into the second cluster. We tested the deviation of F1 scores as a result of over-clustering (+1 and +2 new clusters from optimal clustering) and under-clustering (-1 and -2 clusters from optimal clustering) on synthetic datasets dyn-BF, dyn-BFC, dyn-CY, dyn-LI, dyn-LL and dyn-TF. For each experimental condition of over- or under- clustering, we repeated the process 5 times.

The ideal_edges parameter represents the ideal number of regulators each target gene can have. The maximum number of regulators for a target gene is the total number of TFs in the network (including self-activator if target gene is also a TF). The ideal_edges parameter was screened across different subnetwork density (0 to 1 with 0.1 increment) to test the change in F1 scores. The ideal_edges parameter in relation to subnetwork density was defined as

$$number\ of\ ideal\ edges = \ total\ \#\ of\ TFs \times\ subnetwork\ density$$

The number of generations parameter (num_generations) was screened across different values (100 to 300 with 50 increment) to test the change in F1 scores and run time.

5. Comparisons of OneSC simulator and BoolODE

10 random networks were generated for each unique combinations of network sizes (5, 10, 15, 20) and network density (0.2, 0.4, 0.6, 0.8, 1) using networkx (Hagberg *et al.*, 2008) (version 2.8.8). BoolODE was cloned from https://github.com/Murali-group/BoolODE (commit id: 852b9b5d7e4aa95f9b37a301b551cd09d98b664a). To test the single-core performance, BoolODE simulations were done with no post processing (do_post_processing: false), no parallel (do_parallel: false), 1 core (nClusters: 1), 5 cell simulations (num_cells: 5) and simulation time of 50 (simulation_time: 50). The same was applied for soft-heaviside version of BoolODE. The above parameters gave 5000 distinct simulation steps for each cell simulation. OneSC simulations were ran 5 times with 5000 simulation steps for fair comparisons and runtime was recorded. To benchmark the simulators using parallel, the parallel option is turned on (do_parallel: true) and the number of cores is set to 5 (nclusters: 5) for BoolODE and the soft-heaviside version of BoolODE. OneSC simulations were ran using 5 cores. The benchmark was done on AWS c5.4xlarge EC2 instance running Ubuntu.

To compare the similarity of synthetic cells generated by OneSC and BoolODE, we first downloaded BoolODE generated cells from different gold standard networks (BF, BFC, CY, LI-extended and TF). While BF, BFC, CY, TF were downloaded directly from https://doi.org/10.5281/zenodo.3378975 (5000 cells, sample id 1), LI structure was re-simulated with simulation time of 7 instead of 5 to fully reach the steady state. Same gold standard networks were used for OneSC to generate synthetic cells (100 simulation runs, 2500 simulation steps per run).

To quantify the similarity of gene expression dynamics between the synthetic cells generated by OneSC and those by BoolODE using the same gold standard networks, we first scaled the simulation timesteps so that both sets of synthetic cells have simulation time starting at 0 and ending at 1. Synthetic cells from BoolODE were already categorized into trajectories. We next categorized the OneSC simulated cells from different simulation runs into different trajectories based on the steady states Boolean profiles and averaged the expression profiles from simulations runs with the same steady states. Next, we labelled OneSC generated trajectories by finding the trajectory ID from BoolODE generated data with steady state Boolean profiles that match 100%. To generate the expression dynamics of a gene across one trajectory, all the expression profiles within a 0.01 scaled simulation time bin were averaged for both BoolODE and OneSC generated data. This would generate time-series (100 datapoints at 0.01 interval) of gene expressions across one trajectory. We then calculated the cross-correlations (Derrick,

2004) between the gene expression timeseries from OneSC and BoolODE for each gene and identified the highest cross-correlation (within 0.6 lag) for each gene in a trajectory. For datasets with more than one trajectory, the cross-correlations of gene expressions were counted separately for each trajectory.

To compare cluster similarity, standard single-cell clustering was performed on the synthetic dataset from BoolODE using Scanpy package (Wolf *et al.*, 2018), and the average expression profile was generated for each cluster. Pearson correlations between each synthetic single-cell expression profile from OneSC and cluster average expression profiles from BoolODE were generated. The synthetic cells from OneSC were labelled by the BoolODE cluster id with the highest Pearson correlation.

6. Processing of mouse myeloid progenitors single-cell data

The mouse myeloid progenitors single-cell expression data were from (Paul *et al.*, 2015) and downloaded using Scanpy's data loading function (scanpy.datasets.paul15()). Standard processing including log-normalization, identifying highly variable genes (min_mean = 0.0125, max_mean = 3, min_disp = 0.5), scaling gene expressions (max_value = 10), neighborhood graph construction (n_neighbors = 10, n_pcs = 10), and clustering (leiden cluster with resolution 0.5) were done using Scanpy package (Wolf *et al.*, 2018). The clusters were annotated based on the cluster marker genes. Pseudotime ordering was found using diffusion pseudotime (Haghverdi *et al.*, 2016).

7. Benchmarking inferred myeloid differentiation networks

OneSC and other methods were used to generate myeloid differentiation networks. The inferred networks from all the other methods (with the exception of IQCELL) were converted to concrete Boolean networks by finding the threshold that would result in the same number of edges as OneSC network or less if it exceeds the maximum possible number of edges. All the networks were simulated using OneSC simulator (with the exception of IQCELL) with 1800 time steps and 200 simulation runs using the activity profile of CMP as the initial condition. The default simulation platform for IQCELL was used to simulate its trajectory specific networks. The end point expression profile of each simulation run was binarized (expression threshold of 1) into Boolean activity profiles, and the unique Boolean terminal states for the inferred GRNs were found. Percent agreement was used to measure how similar the real terminal states are to the terminal states generated by inferred networks.

$$percent\ agreement = \frac{\#\ of\ genes\ that\ match\ status}{total\ \#\ of\ genes}$$

The real terminal states (MK, erythrocytes, monocytes, granulocytes) were compared with all the terminal states generated by inferred networks and the maximum percent agreement was assigned to each of the real terminal states as a metric to evaluate whether the inferred network can generate faithful terminal states.

8. Assign cell type annotations to simulated cells

The end point expression profile of each simulation was binarized and compared with the Boolean activity status of the cell type clusters in the real single-cell data. The terminal state of the simulation was assigned to the cell type cluster whose Boolean activity status profile perfectly matches the binarized expression profiles of the simulation's endpoint (percent agreement of 1). If the final state of the simulation does not match with any of the terminal cell state's Boolean activity status profiles, then that simulation's steady state is labelled as "other". The proportions of terminal states reached were calculated for wildtype and perturbation simulations.

In **Supplementary Fig S10** and **Fig 4D**, because there are intermediate synthetic cells that were in transitions, it would not be appropriate use the stringent metric of 100% agreement in Boolean activity status profile matching to annotate cells. Instead, the simulated cells were annotated by assigning the cell type cluster whose Boolean activity status have the lowest Euclidean distance with the simulated expression profiles. The real Boolean activity status profiles were encoded as 0 (off) and 2 (on) to align with the range used in OneSC's simulated expression. In **Fig 4D**, all the simulated cells from simulation runs were labelled as "other" if the terminal states were determined to be "other".

Supplementary Figures

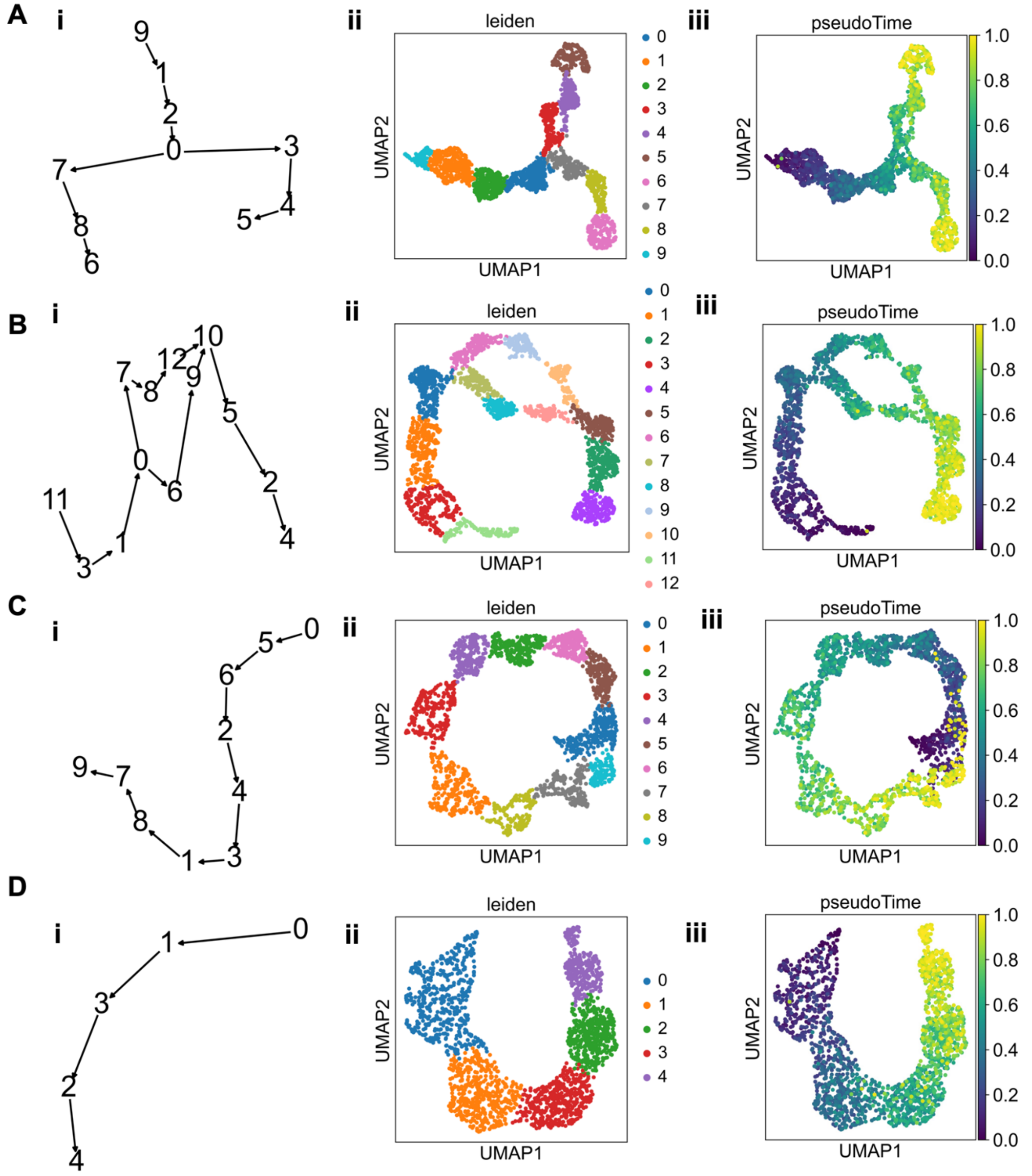

Supplementary Figure 1. Manually curated input of BEELINE synthetic data used by OneSC for gene regulatory network inference. (A) Dyn-BF data's (i) cell state transition graph, (ii) cluster annotation overlayed on UMAP embeddings, and (iii) inferred pseudotime overlayed on UMAP embeddings. (B) Dyn-BFC data's (i) cell state transition graph, (ii) cluster annotation overlayed on UMAP embeddings, and (iii) inferred pseudotime overlayed on UMAP embeddings. (C) Dyn-

CY data's (i) cell state transition graph that was broken up to form a linear structure, (ii) cluster annotation overlayed on UMAP embeddings, and (iii) inferred pseudotime overlayed on UMAP embeddings. (D) Dyn-LI data's (i) cell state transition graph, (ii) cluster annotation overlayed on UMAP embeddings, and (iii) inferred pseudotime overlayed on UMAP embeddings.

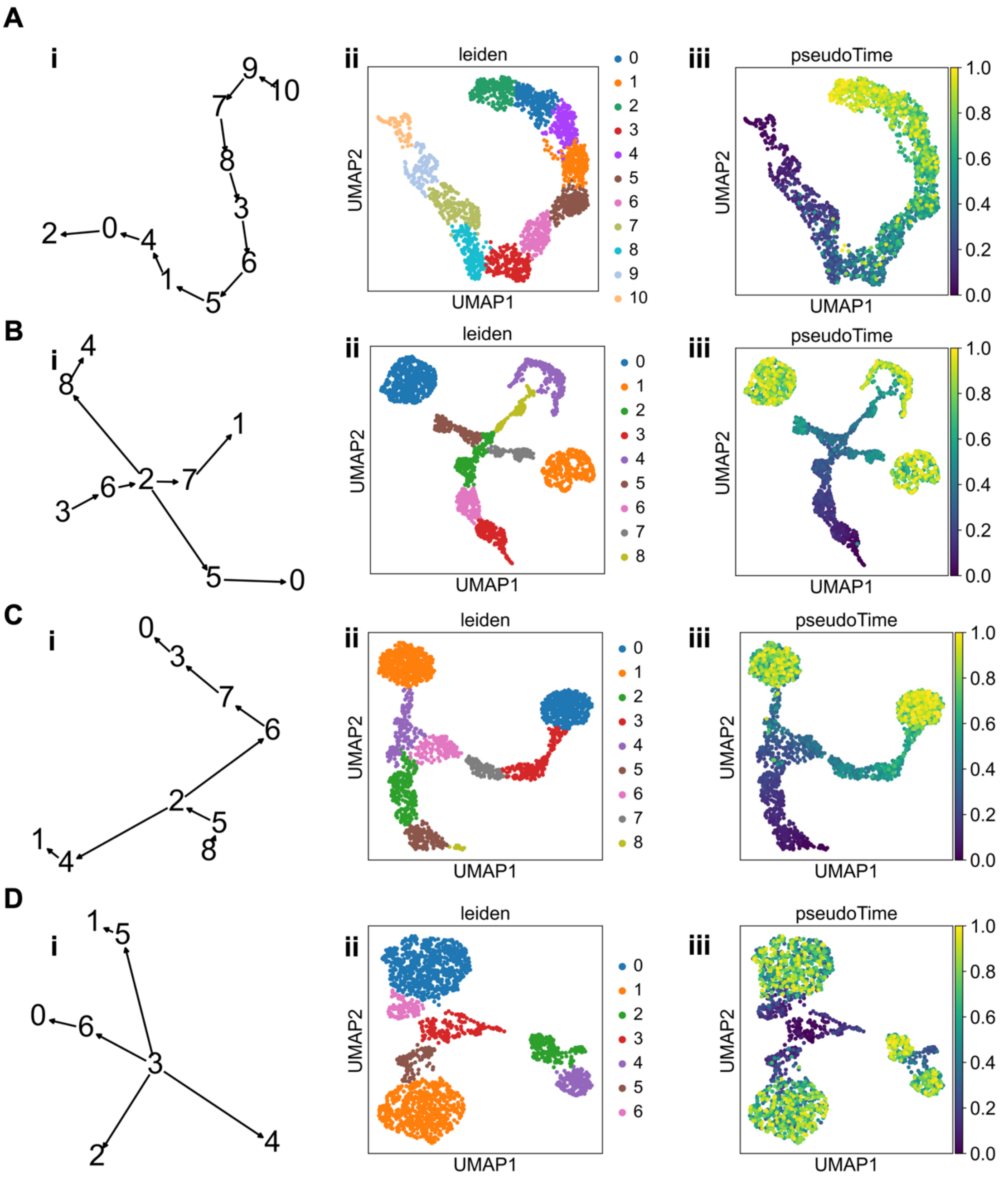

Supplementary Figure 2. Manually curated input of BEELINE synthetic data used by OneSC for gene regulatory network inference. (A) Dyn-LL data's (i) cell state transition graph, (ii) cluster annotation overlayed on UMAP embeddings, and (iii) inferred pseudotime overlayed on UMAP embeddings. (B) Dyn-TF data's (i) cell state transition graph, (ii) cluster annotation overlayed on UMAP embeddings, and (iii) inferred pseudotime overlayed on UMAP embeddings. (C) GSD data's (i) cell state transition graph, (ii) cluster annotation overlayed on UMAP embeddings, and (iii) inferred pseudotime overlayed on UMAP embeddings. (D) HSC data's (i) cell state transition

graph, (ii) cluster annotation overlayed on UMAP embeddings, and (iii) inferred pseudotime overlayed on UMAP embeddings.

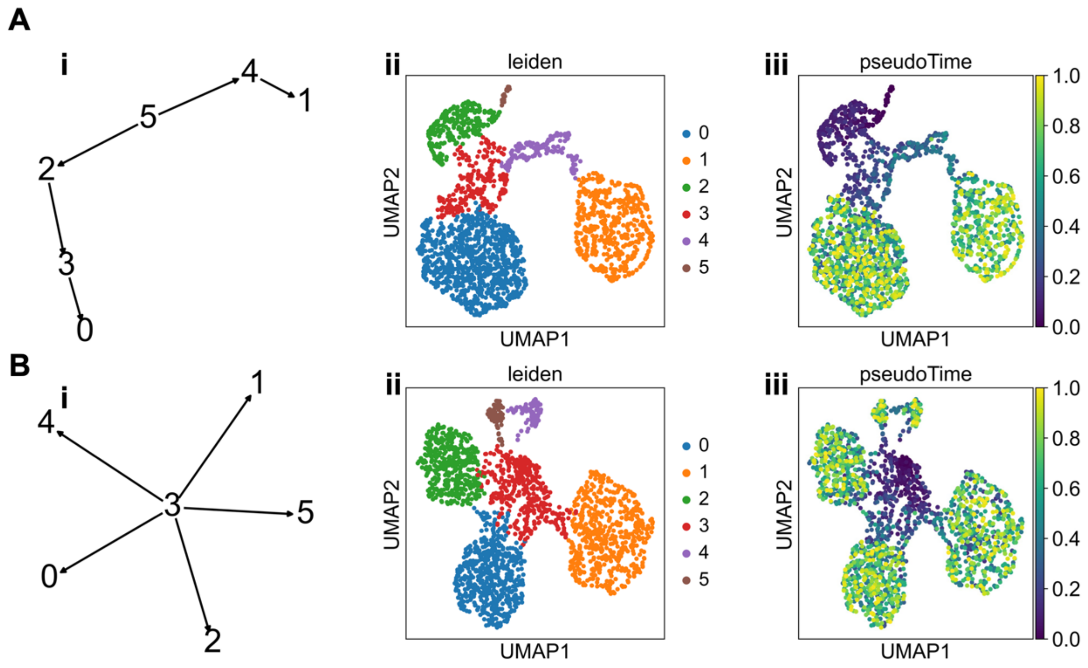

Supplementary Figure 3. Manually curated input of BEELINE synthetic data used by OneSC for gene regulatory network inference. (A) mCAD data's (i) cell state transition graph, (ii) cluster annotation overlayed on UMAP embeddings, and (iii) inferred pseudotime overlayed on UMAP embeddings. (B) VSC data's (i) cell state transition graph, (ii) cluster annotation overlayed on UMAP embeddings, and (iii) inferred pseudotime overlayed on UMAP embeddings.

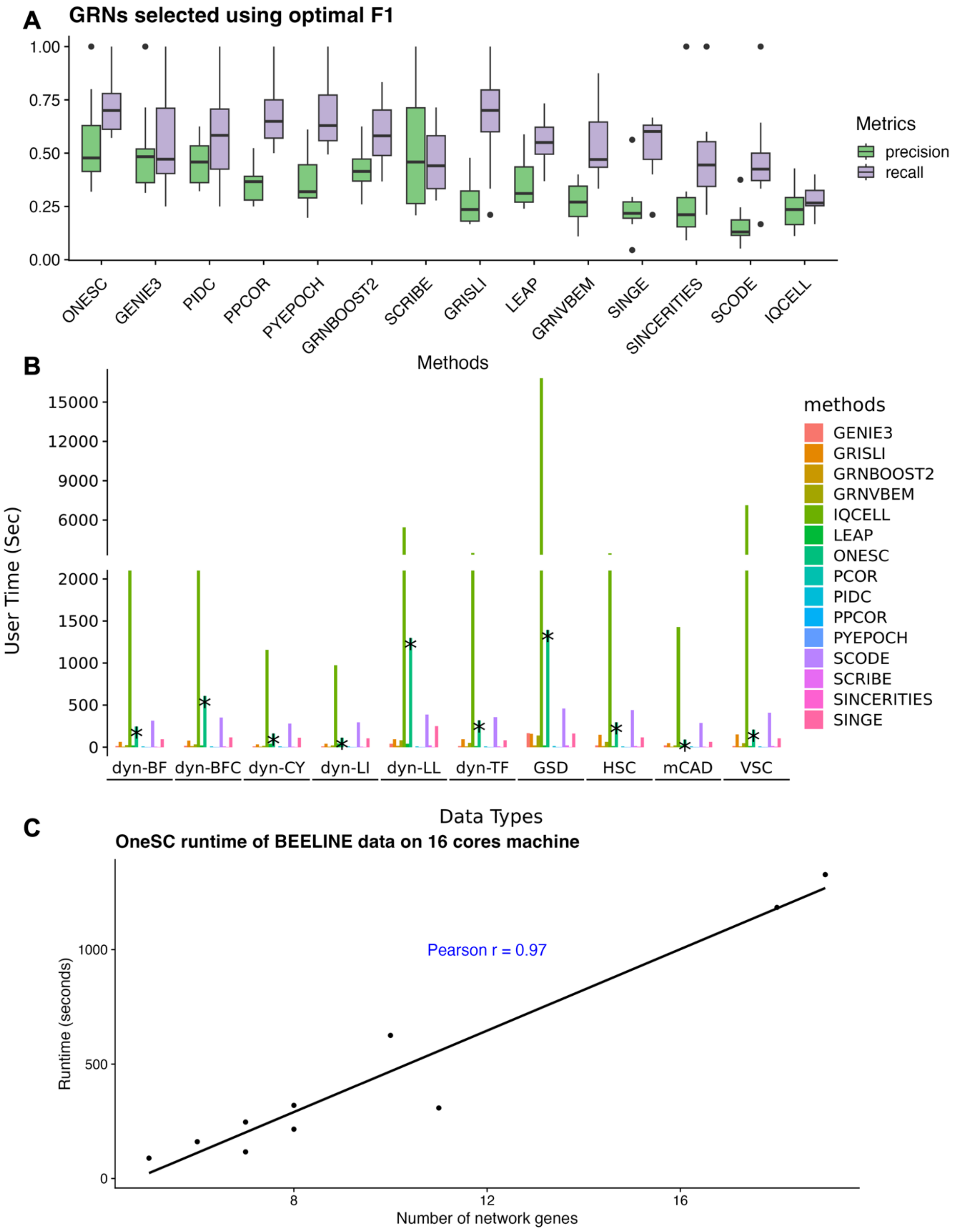

Supplementary Figure 4. Benchmarking OneSC with other gene regulatory network inference methods using the BEELINE platform. (A) Precision and recall scores of OneSC inferred networks and F1 maximized networks from other gene regulatory network inference methods

tested across 10 BEELINE synthetic datasets (dyn-LI, dyn-LL, dyn-BF, dyn-BFC, dyn-TF, dyn-CY, mCAD, VSC, HSC, GSD). (B) Runtime of gene regulatory network inference methods tested on 10 BEELINE synthetic datasets. * indicates the runtime for OneSC. All benchmarks were done on c5.4xlarge AWS EC2 instance (16 CPU cores). (C) Scatter plot showing the correlation between OneSC network inference's runtime and the number of genes in the network.

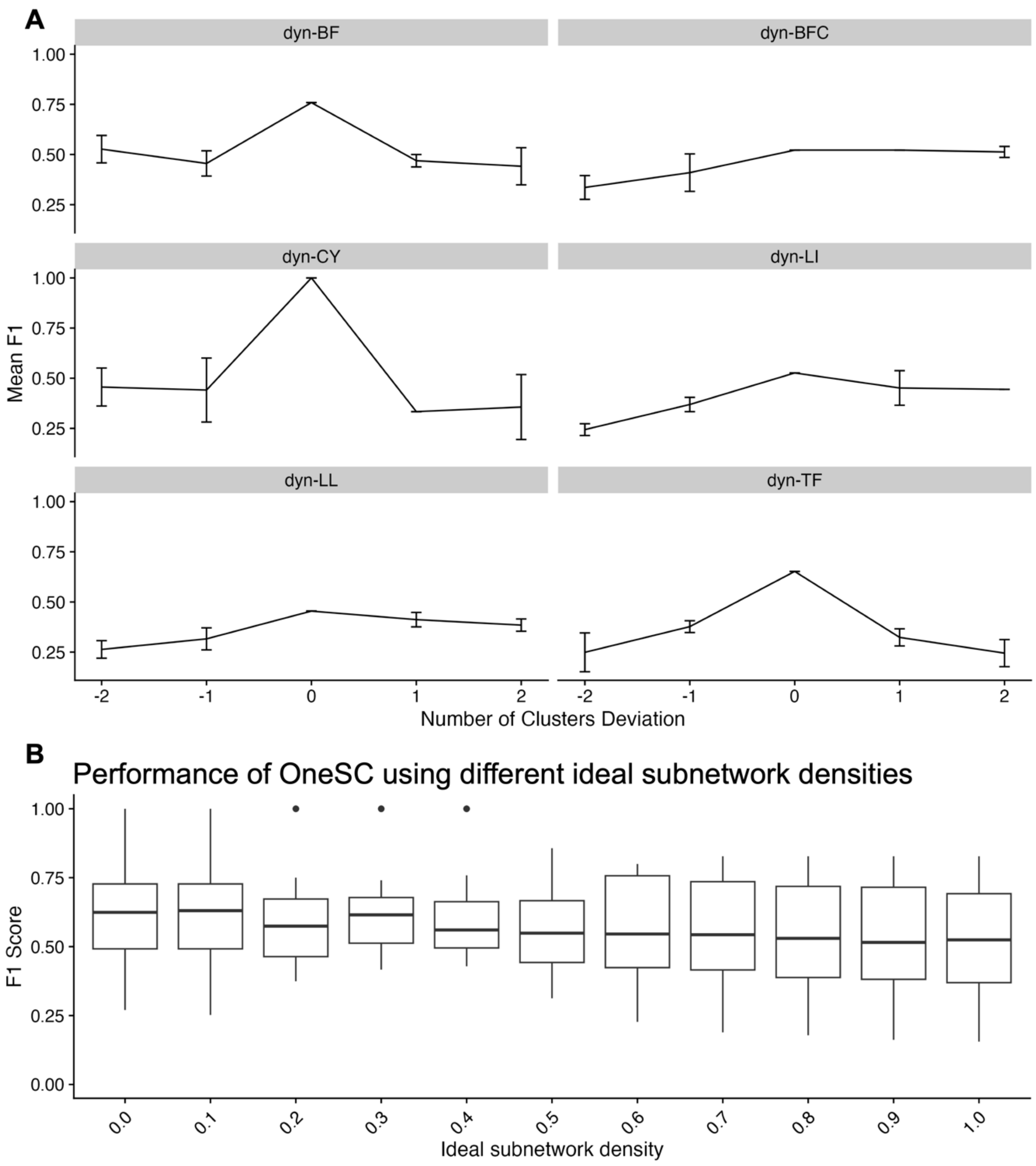

Supplementary Figure 5. Parameter sensitivity testing of OneSC. (A) Line plots showing the changes in average F1 scores when there is a deviation (-2, -1, +1, +2) in the clustering through the artificial splitting or merging of optimal clusters that were used by OneSC in the benchmark against other GRN inference methods. There are 5 trials per cluster deviation. The error bars represent standard deviations in F1 scores. (B) Box plot of F1 scores of OneSC networks inferred from 10 BEELINE synthetic datasets (dyn-LI, dyn-LL, dyn-BF, dyn-BFC, dyn-TF, dyn-CY, mCAD, VSC, HSC, GSD) using different ideal subnetwork densities. The ideal subnetwork

density can be converted to ideal number of subnetwork regulatory edges parameter for OneSC by multiplying the ideal subnetwork density with the total number of potential regulators.

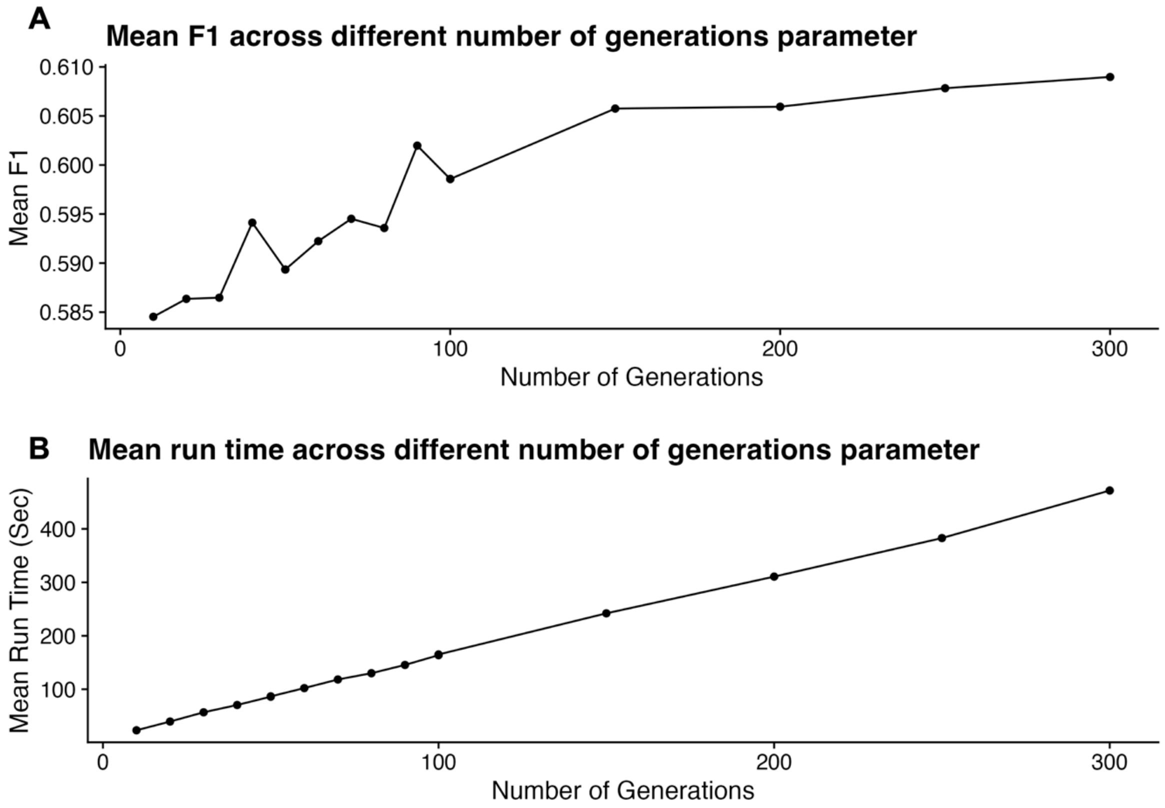

Supplementary Figure 6. Testing the performance of OneSC using different number of generations for genetic algorithm. (A) Line plot showing the average F1 scores of OneSC tested on 10 BEELINE synthetic datasets (dyn-LI, dyn-LL, dyn-BF, dyn-BFC, dyn-TF, dyn-CY, mCAD, VSC, HSC, GSD) using different parameters of genetic algorithm generations. (B) Line plot showing the average run time of OneSC tested on 10 BEELINE synthetic datasets using different numbers of genetic algorithm generations.

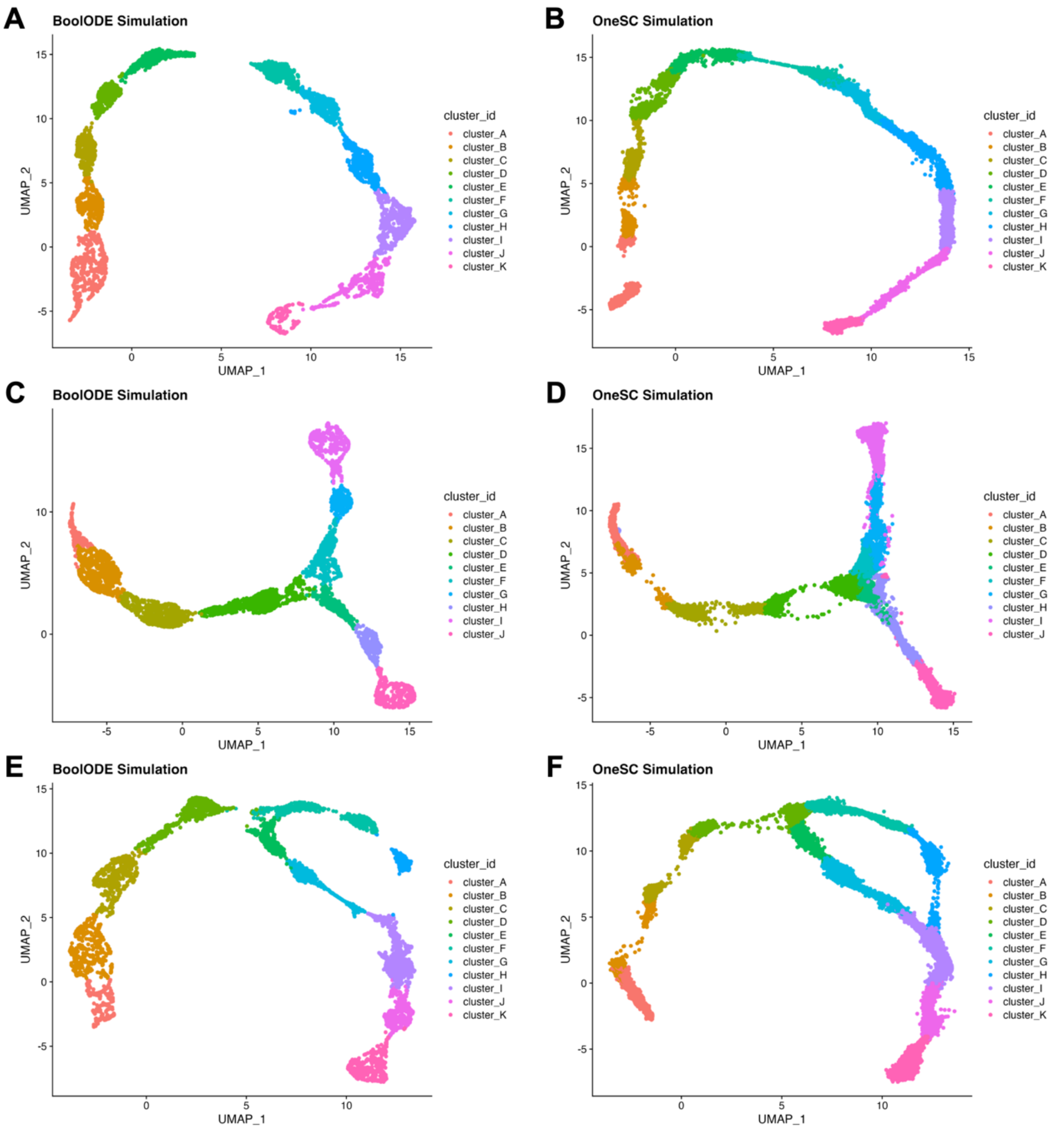

Supplementary Figure 7. Comparison of synthetic single-cell data simulated from OneSC and BoolODE. The cluster assignments for BoolODE synthetic data were done using standard Scanpy clustering. The cluster assignment for each cell in the OneSC synthetic data were labelled via finding the BoolODE cell cluster with the highest Pearson correlation in expression profiles. UMAPs showing synthetic data generated from dyn-LI gold standard network by (A) BoolODE and (B) OneSC. UMAPs showing synthetic data generated from dyn-BF gold standard network by (C) BoolODE and (D) OneSC. UMAPs showing synthetic data generated from dyn-BFC gold standard network by (E) BoolODE and (F) OneSC.

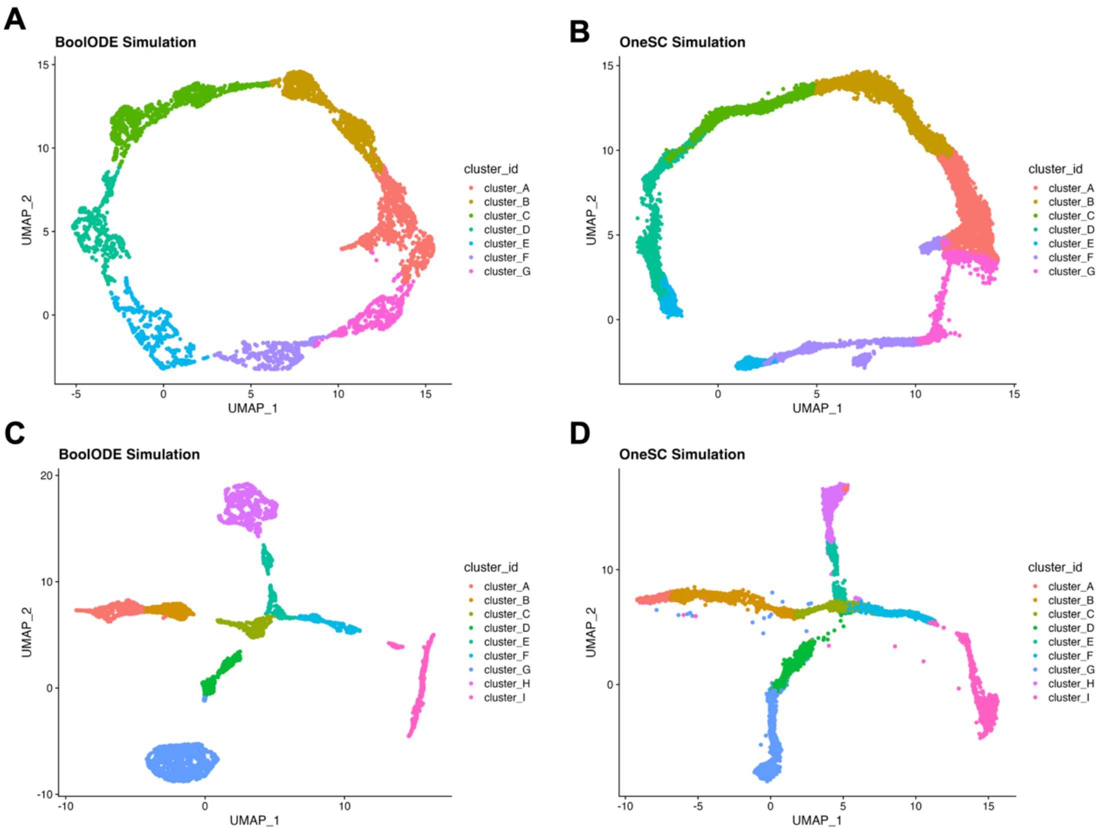

Supplementary Figure 8. Comparison of synthetic single-cell data simulated from OneSC and BoolODE. The cluster assignments for BoolODE synthetic data were done using standard Scanpy clustering. The cluster assignment for each cell in the OneSC synthetic data were labelled via finding the BoolODE cell cluster with the highest Pearson correlation in expression profiles. UMAPs showing synthetic data generated from dyn-CY gold standard network by (A) BoolODE and (B) OneSC. UMAPs showing synthetic data generated from dyn-TF gold standard network by (C) BoolODE and (D) OneSC.

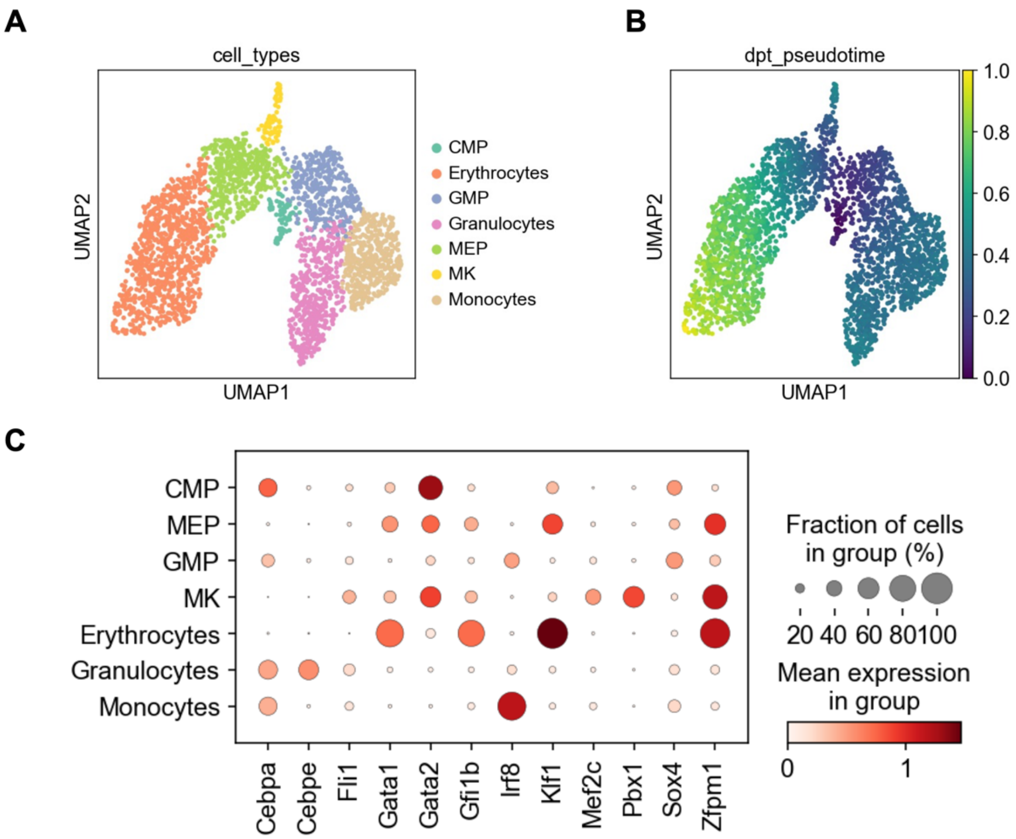

Supplementary Figure 9. Preprocessing of Paul et al myeloid progenitor dataset. (A) UMAP showing the cell type cluster annotations. (B) UMAP showing the diffusion pseudotime assignments. (B) Dot plot showing the 12 dynamically expressed transcription factors across different cell types.

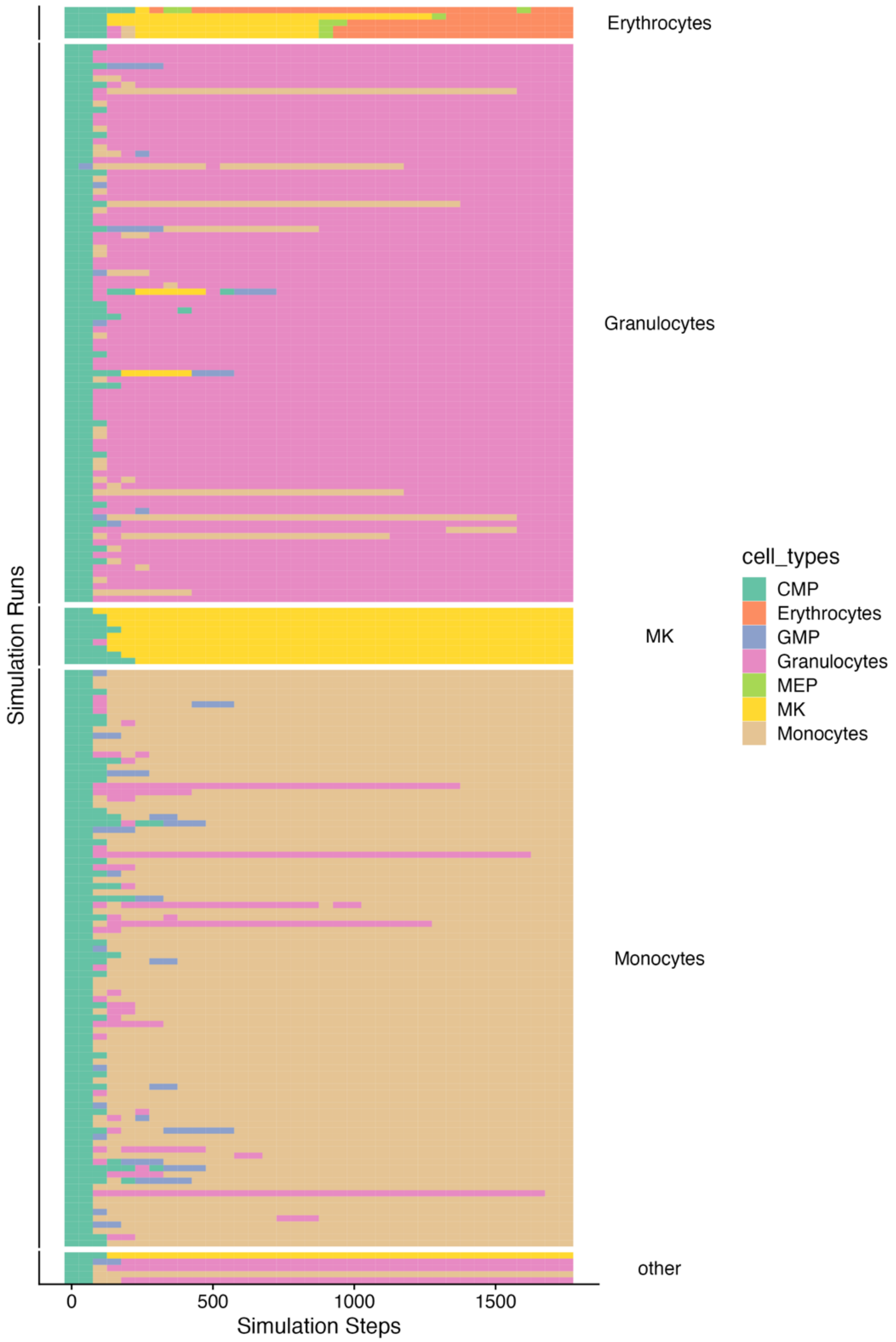

Supplementary Figure 10. Heatmap showing the cell type assignment of 200 OneSC simulation runs from inferred network to capture myeloid differentiation trajectories. Each time step (a synthetic cell) in the simulation run is assigned to the cell type with the lowest distance in Boolean activity profiles.

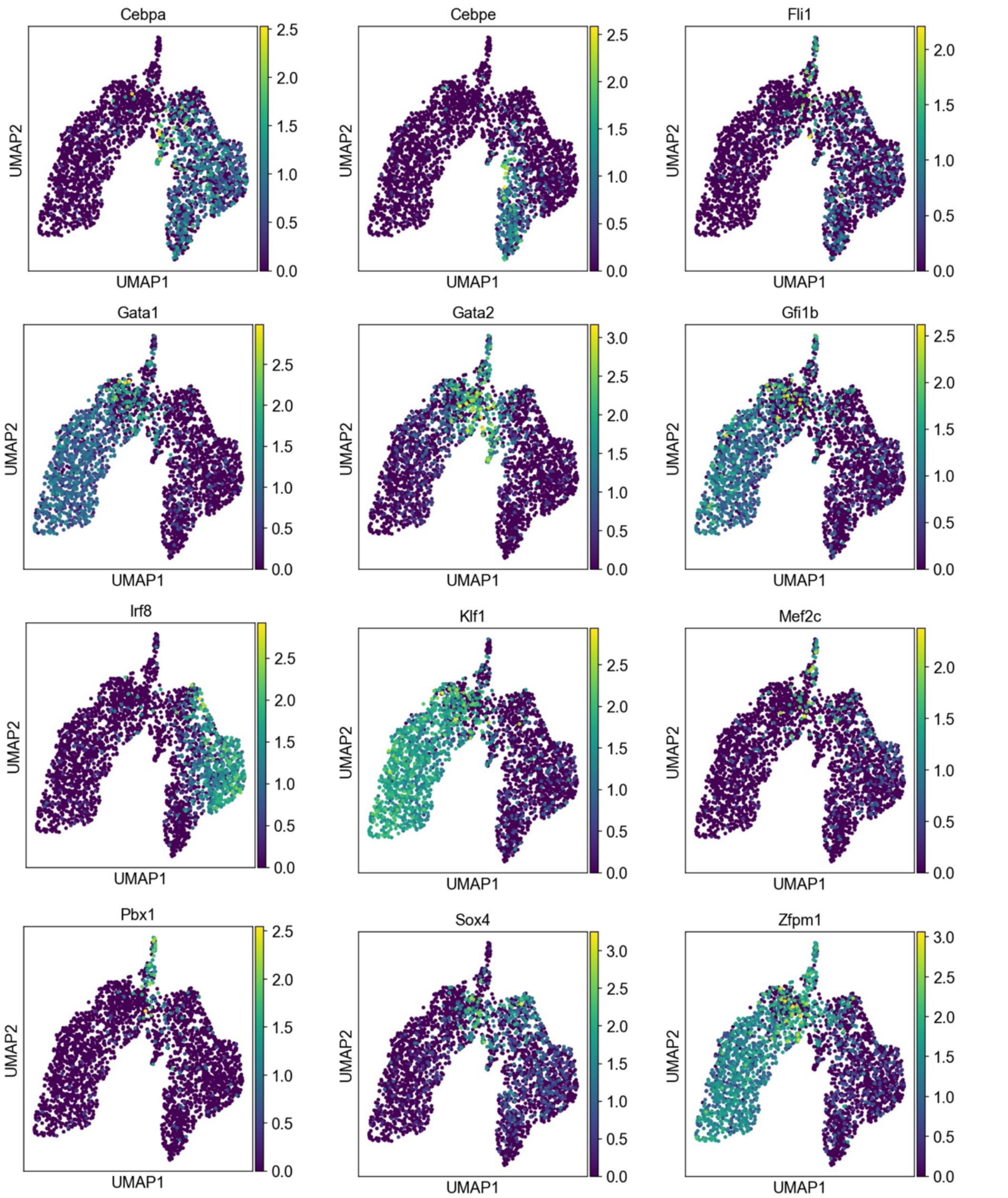

Supplementary Figure 11. UMAPs showing the normalized expression values of the 12 dynamically expressed transcription factors in the Paul et al myeloid datasets.

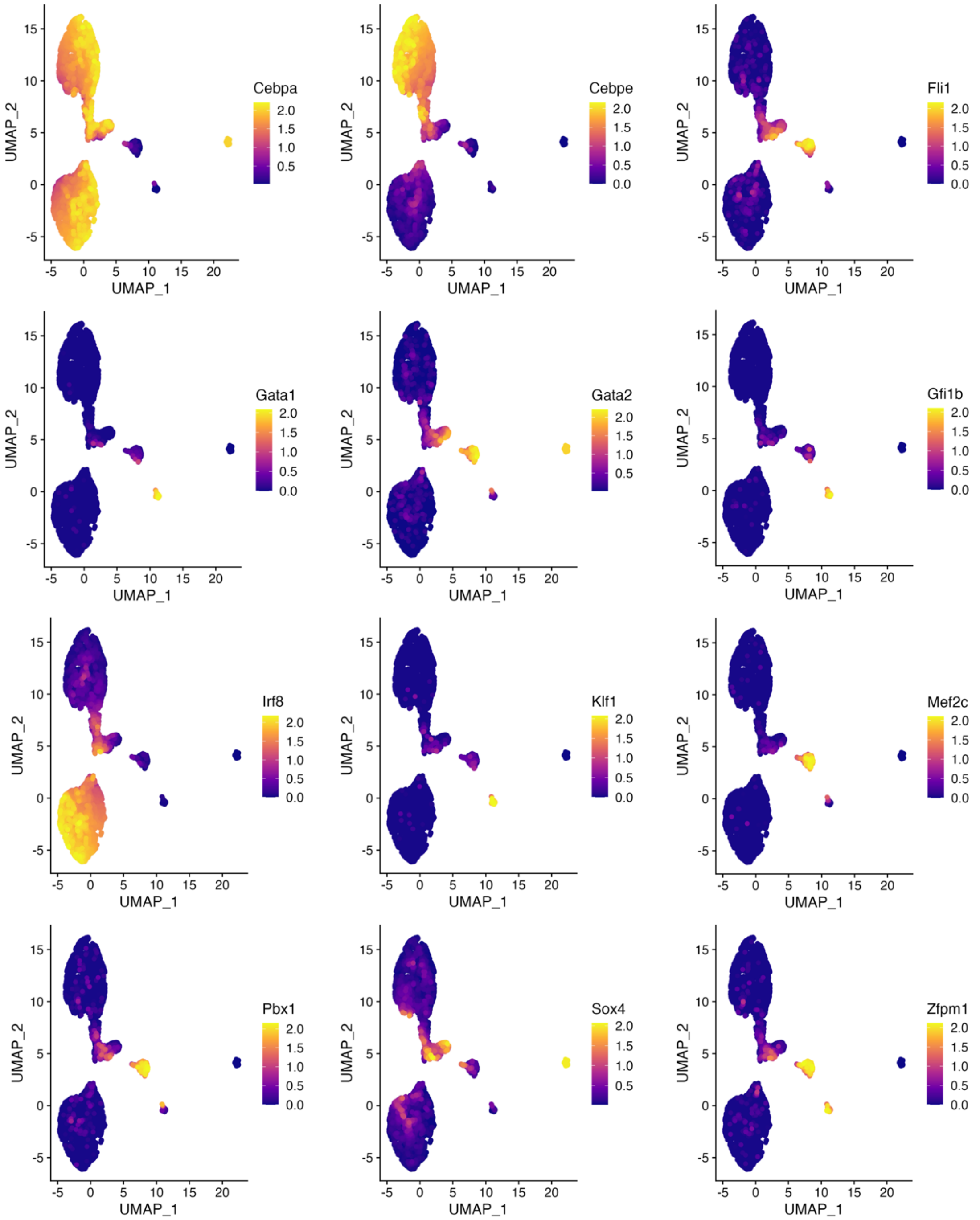

Supplementary Figure 12. UMAPs showing the expression values of the 12 dynamically expressed transcription factors in the OneSC simulated cells.

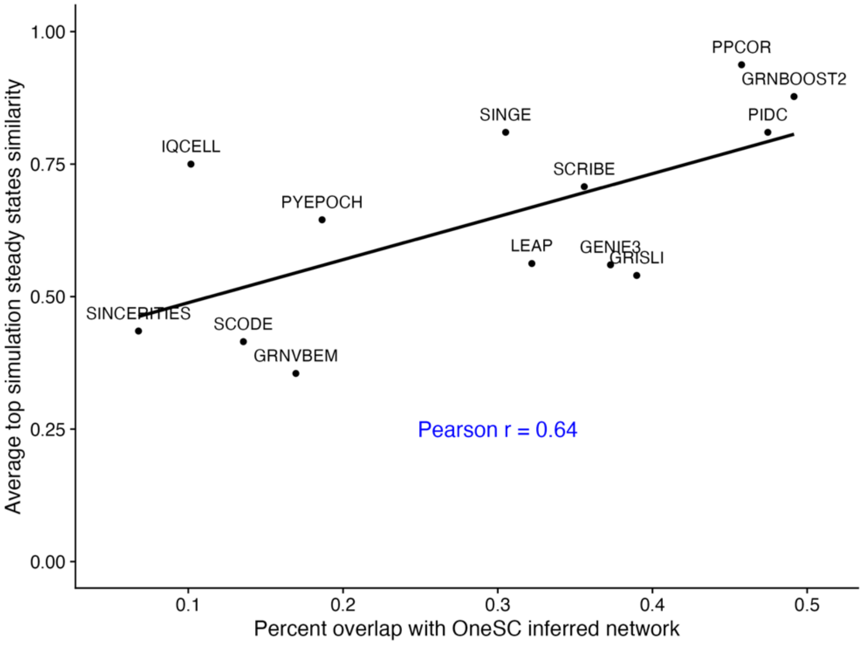

Supplementary Figure 13. Scatter plot showing the correlation of the percent overlap of regulatory edges between other inferred myeloid differentiation networks and OneSC inferred myeloid differentiation network, and the average similarity of the simulation steady states to real steady states.

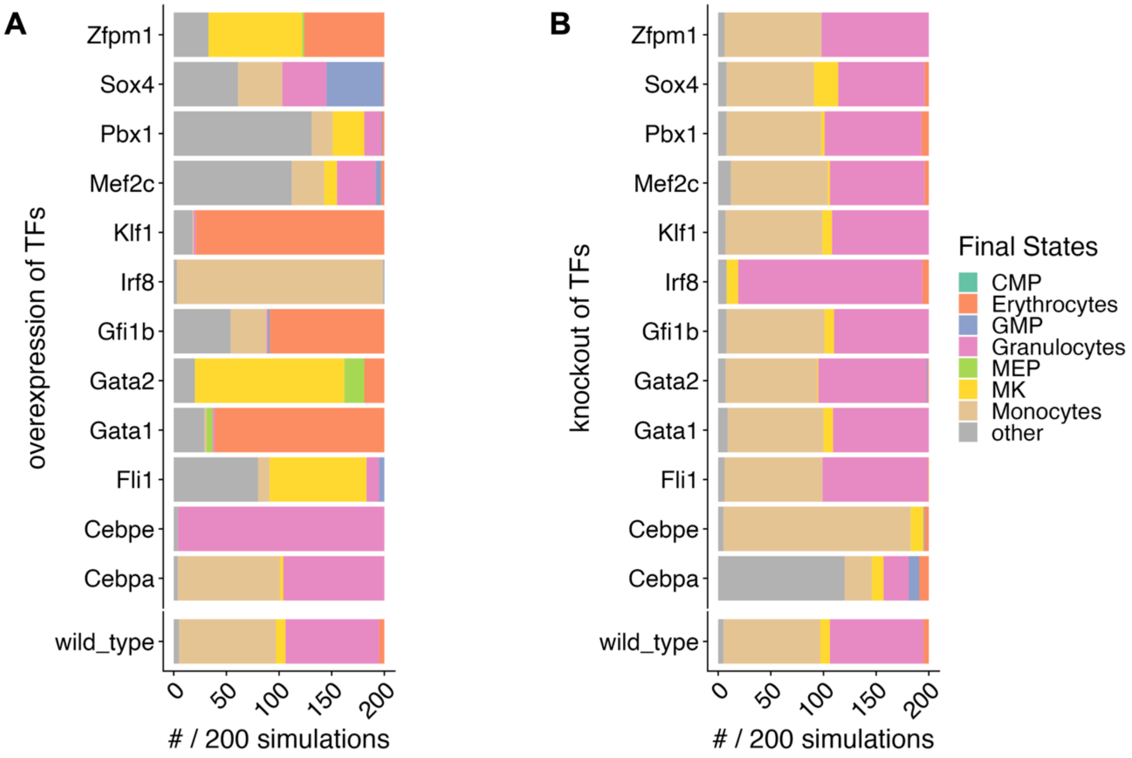

Supplementary Figure 14. (A) Barplot showing the proportion of steady states from OneSC's overexpression simulations of the 12 dynamically expressed TFs and no perturbation simulation (wild_type). (B) Barplot showing the proportion of steady states from OneSC's knockout simulations of the 12 dynamically expressed TFs and no perturbation simulation (wild_type).

Supplementary Table

Supplementary Table 1. Differentially expressed genes for cell type clusters in Paul et al dataset.

References

Albadr,M.A. *et al.* (2020) Genetic algorithm based on natural selection theory for optimization problems. *Symmetry*, **12**, 1758.

Bessho,Y. (2003) Oscillations, clocks and segmentation. *Curr. Opin. Genet. Dev.*, **13**, 379–384.

Chen,J. *et al.* (2005) Negative feedback loop formed by Lunatic fringe and Hes7 controls their oscillatory expression during somitogenesis. *Genesis*, **43**, 196–204.

Derrick,T.R. (2004) Time Series Analysis: The Cross-Correlation Function. In *Innovative Analyses of Human Movement* (ed. N.Stergiou), pp. 189-205. Champaign, IL: Human Kinetics Publishers.

Gad,A.F. (2021) PyGAD: An Intuitive Genetic Algorithm Python Library. *arXiv*.

Hagberg,A.A. *et al.* (2008) Exploring Network Structure, Dynamics, and Function using NetworkX. SciPy 2008.

Haghverdi,L. *et al.* (2016) Diffusion pseudotime robustly reconstructs lineage branching. *Nat. Methods*, **13**, 845–848.

Jalihal,A.P. (2020) Mathematical modeling of macronutrient signaling in Saccharomyces cerevisiae. Doctoral dissertation. Virginia Polytechnic Institute and State University. http://hdl.handle.net/10919/99306

Katoch,S. *et al.* (2021) A review on genetic algorithm: past, present, and future. *Multimed. Tools Appl.*, **80**, 8091–8126.

Kloeden,P.E. and Platen,E. (1992) Numerical solution of stochastic differential equations Springer Berlin Heidelberg, Berlin, Heidelberg.

Momiji,H. and Monk,N.A.M. (2008) Oscillatory expression of Hes family transcription factors: insights from mathematical modelling. *Adv. Exp. Med. Biol.*, **641**, 72–87.

Müssel,C. *et al.* (2010) BoolNet--an R package for generation, reconstruction and analysis of Boolean networks. *Bioinformatics*, **26**, 1378–1380.

Paul,F. *et al.* (2015) Transcriptional heterogeneity and lineage commitment in myeloid progenitors. *Cell*, **163**, 1663–1677.

Pratapa,A. *et al.* (2020) Benchmarking algorithms for gene regulatory network inference from single-cell transcriptomic data. *Nat. Methods*, **17**, 147–154.

Shannon,C.E. (1938) A symbolic analysis of relay and switching circuits. *Trans. Am. Inst. Electr. Eng.*, **57**, 713–723.

Su,E.Y. *et al.* (2022) Reconstruction of dynamic regulatory networks reveals signaling-induced topology changes associated with germ layer specification. *Stem Cell Reports*, **17**, 427–442.

Trapnell,C. *et al.* (2014) The dynamics and regulators of cell fate decisions are revealed by pseudotemporal ordering of single cells. *Nat. Biotechnol.*, **32**, 381–386.

Wolf,F.A. *et al.* (2018) SCANPY: large-scale single-cell gene expression data analysis. *Genome Biol.*, **19**, 15.
